# Natural Product Target Identification of Wheldone, a Fungal Metabolite, as a KIF11 Inhibitor in Ovarian Cancer Using the DiffPOP (Differential Protein Precipitation) Method

**DOI:** 10.1016/j.mcpro.2026.101558

**Published:** 2026-03-23

**Authors:** Manead Khin, Alejandra Cavazos Saldana, Manuel Rangel-Grimaldo, Huzefa A. Raja, Daniel Abegg, Julia Ekiert, Chang Liu, Sweta Misra, Kiira Ratia, Alexander Adibekian, Yu Gao, Samuel K. Kulp, Christopher C. Coss, Nicholas H. Oberlies, Joanna E. Burdette

**Affiliations:** 1 Department of Pharmaceutical Sciences, University of Illinois at Chicago, Chicago, Illinois, USA; 2 Department of Chemistry and Biochemistry, University of North Carolina at Greensboro, Greensboro, North Carolina, USA; 3 Department of Chemistry, University of Illinois at Chicago, Chicago, Illinois, USA; 4 Division of Pharmaceutics and Pharmacology, College of Pharmacy, The Ohio State University, Columbus, Ohio, USA

## Abstract

Wheldone, a fungal metabolite, was identified as a cytotoxic compound in high-grade serous ovarian cancer (HGSOC). Wheldone induced caspase 3/7–dependent apoptosis and reduced migration, invasion, and spheroid growth. Wheldone stimulated apoptosis in chemoresistant HGSOC models. Wheldone treatment caused significant downregulation of HNRNPD, a DNA repair protein, and increased DNA damage that could be blocked by *N*-acetyl-L-cysteine. *In vivo*, wheldone displayed minimal toxicity but was rapidly cleared from circulation, despite *in vitro* metabolic stability. Wheldone treatment *in vivo* did not demonstrate significant reduction in tumor burden. Therefore, in order to overcome these liabilities, it was necessary to find the protein target of wheldone so that modifications can be made to improve the drug-like characteristics of the compound. Using the drug-target interaction proteomics method, differential precipitation of proteins, wheldone was found to act as an inhibitor of Kinesin superfamily protein 11 (KIF11), a motor protein essential for mitotic spindle formation. An ATPase biochemical cell-free assay confirmed direct binding and functional inhibition of KIF11. Wheldone resulted in G2/M arrest and downstream regulation of mitotic proteins such as TPX2, AURKA, and phospho-histone H3. Proteomics after treatment of wheldone in four different HGSOC cancer cell lines all supported changes consistent with mitotic spindle assembly disruption. Further, KIF11 was one of only 13 proteins upregulated in all 4 cell lines treated. Overall, wheldone was found to be a fungal metabolite that inhibits KIF11 in chemoresistant ovarian cancer, with future studies needed to improve its pharmacokinetics and delivery.

## Highlights

• Wheldone is a fungal metabolite that inhibits KIF11.
• DiffPOP method can identify protein binding targets of unlabeled small molecules.
• Kiff 11 inhibitors induce apoptosis in high grade serous ovarian cancer cells.

## Graphical Abstract

**Figure:**
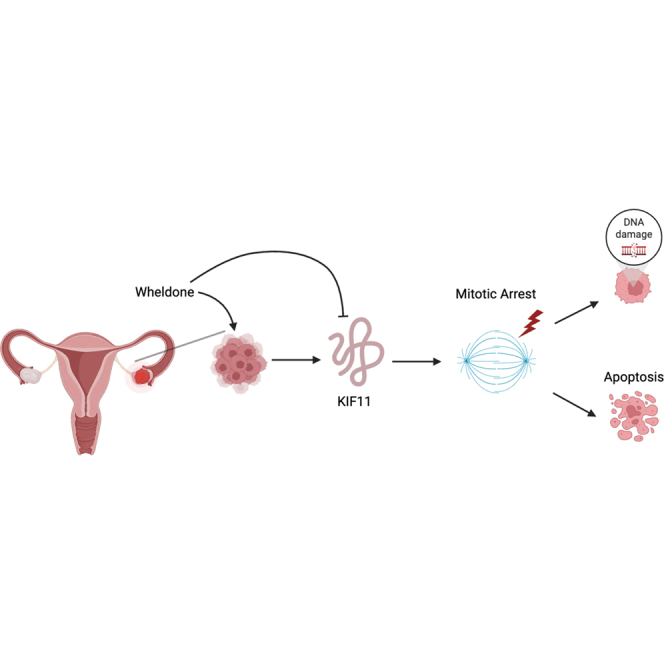

## In Brief

DiffPOP proteomics was used to identify the target of a fungal metabolite, wheldone. Wheldone was confirmed to induce cell death in high grade serous cancer cell lines by inhibiting KIF11.

High-grade serous ovarian cancer (HGSOC) is the deadliest ovarian cancer, with a ∼50% 5-years survival rate (https://seer.cancer.gov/statfacts/html/ovary.html). Despite treatment, over 80% of patients relapse within 24 months (1), with >90% mortality for chemoresistant cases (2). Current therapies, such as anti-angiogenic agents and Poly(ADP-ribose) polymerase inhibitors, offer low overall survival (3), highlighting the need for novel chemotherapeutics.

Natural products are a valuable reservoir of bioactive scaffolds that have inspired the development of anticancer agents (4). Wheldone, a structurally unique fungal metabolite (5), demonstrated cytotoxic activity against various cancer cell lines (6). It was generated via the fungal-fungal coculture of *Aspergillus fischeri* and *Xylaria flabelliformis*, with the premise being that the competition for nutrients would stimulate otherwise silent biosynthetic gene clusters and produce new chemistry (7). However, the mechanism of action of wheldone in HGSOC remains unknown, and this study investigates the *in vitro* and *in vivo* activity in HGSOC, along with the molecular target of this fungal metabolite.

One component of studying mechanism of action for natural products is identifying the cellular protein targets that interact with the compounds, and this information can be critical when attempting to advance leads into more clinical settings. Identification of protein binding for natural products can explain their biological activity in cancer cells. Additionally, this can help explain potential toxicities or prioritize which types of cancers should be treated with a specific molecule based on expression patterns. Affinity based methods typically rely on synthetic or semi-synthetic modifications, which require knowledge of the target of interaction to avoid modifying the compound in a particularly important motif. However, natural products are often difficult to synthesize, present in small quantities, and without known targets to inform the design of modifications to incorporate tags or handles for immobilization or labeling. Currently, there are several unlabeled methods including drug affinity responsive target stability, limited proteolysis, solvent induced precipitation, and Cellular Thermal Shift Assay. The Differential Precipitation of Proteins (DiffPOP) method was originally developed by the Yates lab at Scripps Research, and allows for target profiling under physiologically relevant conditions, which may provide a strong method for investigating protein targets of natural products, which was explored in this project (8).

Kinesin superfamily proteins (KIFs) are essential motor proteins for cellular processes, such as cellular processes, including cell division, intracellular transport, and organelle positioning. KIF11 (kinesin-5/Eg5) is crucial during mitosis, utilizing ATP hydrolysis to move along microtubules, ensuring proper genetic segregation. By utilizing the energy from ATP hydrolysis, KIF11 undergoes conformational changes that allow it to move along microtubules. This stepwise movement is essential in ensuring the successful segregation of genetic material into daughter cells, making KIF11 indispensable for cell division. Its elevated expression has been observed in various cancers, including ovarian cancer, where KIF11 plays a pivotal role in driving uncontrolled cell growth. Inhibiting KIF11 causes mitotic arrest and apoptosis in proliferating cancer cells, making it an anticancer target (9, 10, 11, 12).

Small molecule KIF11 inhibitors are a therapeutic strategy, with some in clinical trials for various cancers (10, 13). Preclinical studies show effectiveness (11, 14), but clinical trials for KIF11 inhibitors have been largely unsuccessful, providing limited benefits and no long-term survival advantage, underscoring the need for further research (15). Hence, this study identified wheldone as a KIF11 inhibitor. Importantly, the structure of wheldone does not resemble the existing KIF11 inhibitors, offering the potential for improved structural modification, which is greatly facilitated by confirming the interaction of wheldone with KIF11 as revealed by the DiffPOP method, a proteomics approach that enables differentiation of binding-induced population shifts in protein conformations (16). Our studies identified direct binding and inhibition of the KIF11 protein as well as downstream signaling consistent with KIF11 inhibition, such as G2/M cell cycle arrest, changes in the expression levels of other mitotic proteins, and induction of apoptosis. The application of DiffPOP, thus, provides mechanistic evidence for direct engagement between wheldone and KIF11, strengthening the rationale for its further optimization as a novel inhibitor scaffold.

## Experimental Procedures

### Compound Isolation

Wheldone was isolated and elucidated from cocultures of *A. fischeri* (strain NRRL 181) and *X. flabelliformis* (strain G536) and was >95% pure as monitored by both mass spectrometry and 1H NMR spectroscopy data (Supplemental Figs. S5 and S6) (5, 6).

### Cell Culture

OVCAR3 and OVCAR8 cells were obtained from American Type Culture Collection. OV81.2, OV81.2 CP40, OV231, and OV231 CP30 were gifted by Analisa DiFeo (University of Michigan); OVCAR8-RFP by Sharon Stack (University of Notre Dame); and FT190 by Ronny Drapkin (University of Pennsylvania). OVCAR3 was cultured in RPMI 1640 with 20% FBS, 1% penicillin/streptomycin (P/S), and 10 μg/ml insulin. FT190 was grown in Advanced DMEM/F12 with 20% FBS and 1% P/S. All other cancer cell lines were maintained in DMEM with 10% FBS and 1% P/S. Cells were maintained at 37 °C, 5% CO_2_, validated by short tandem repeat analysis, and confirmed mycoplasma-free in 2024.

### Cell Viability Assay

Cells were seeded in 96-well, clear, flat-bottomed plates at 5000 cells per well, and allowed to attach for 24 h. Compounds (0.1% DMSO concentration) suspended in DMSO were diluted to final concentrations, added to the cells, and incubated. Cell viability was assessed using CellTiter-Blue (Promega) on a Synergy Mx plate reader (BioTek). Data were normalized to vehicle controls, and IC_50_ values were calculated using GraphPad Prism (https://www.graphpad.com/).

### Two-Dimensional Foci Assay

OVCAR3 and OVCAR8 (200 cells per 60 mm dish) were plated, attached for 24 h, and treated with compounds or DMSO for 72 h. Colonies were grown in drug-free media with changes every 3 days. By day 20, foci were fixed (4% paraformaldehyde), stained (0.05% crystal violet), imaged (FluorChem E, ProteinSimple), and quantified using ImageJ (NIH; https://imagej.net/). Counts were normalized to vehicle, and statistical significance was assessed by one-way ANOVA with Dunnett’s test.

### Immunoblot Analysis

Cells were lysed in RIPA buffer (50 mmol/L Tris, pH 7.6, 150 mmol/L NaCl, 1% Triton X-100, 0.1% SDS) with protease (Roche Applied Science, #11697498001) and phosphatase (Sigma, #P0044) inhibitors, incubated at −80 °C, and clarified by centrifugation. Protein concentration was measured via Bradford assay (Bio-Rad #5000205), and samples were resolved by SDS-PAGE and transferred to nitrocellulose. Membranes were blocked in 5% milk/TBST, probed with primary antibodies (1:1000; Supplemental Table S1), and incubated with anti-rabbit secondary (CST #7074, 1:1000). Signal was visualized using SuperSignal West Femto (Thermo Fisher Scientific #34096) and imaged on a FluorChem E system (ProteinSimple).

### Immunofluorescence Assay

Cells (1 × 10^5^) were seeded on 18 mm glass coverslips (Thermo Fisher Scientific #12-542-006) in 6-well plates and incubated overnight. After treatment (≤0.25% DMSO), cells were fixed with 4% paraformaldehyde (Sigma #158127), permeabilized with 0.2% Triton X-100 (Sigma #T9284), and blocked in 1% BSA (Sigma #A4612). Primary antibodies (1:1000; Supplemental Table S1) were applied for 1 h, followed by washes and incubation with secondary antibody (CST #7074, 1:1000) for 1 h. Nuclei were stained with DAPI (0.1 μg/ml, Thermo Fisher Scientific #EN62248), and coverslips were mounted using VECTASHIELD (Vector Labs #H-1000). Images were captured using a Nikon Eclipse E600 (40×) with DS-Ri1 camera and NIS Elements software (microscope.healthcare.nikon.com/en_AOM/products/software/nis-elements) or on a Zeiss LSM 710 confocal microscope (40 × water objective, ZEN software, zeiss.com/microscopy/us/products/software/zeiss-zen.html).

### Annexin V/Propidium Iodide Staining

Cells (OVCAR3, OVCAR8) were seeded at 10,000 cells per flask into T25 flasks, allowed to attach overnight, and treated with compounds for 24 or 48 h. Cells were subjected to an Annexin V–FITC/Propidium Iodide Apoptosis Assay (Nexcelom Biosciences) according to instructions provided by the manufacturer. Fluorescence was detected using a K2 Cellometer (Nexcelom Biosciences). Data were analyzed with the FCS Express program (De Novo, denovosoftware.com). Each replicate contained at least 2000 cells for quantification of fluorescent signal.

### Caspase 3/7 Reporter Assay

Cells were seeded in 96-well, clear, white-walled plates at 5000 cells per well and allowed to attach overnight. Compounds, staurosporine (Selleckchem, #S1421), and ZVAD-FMK (Promega, #G7231) suspended in DMSO were diluted to final concentrations and added to the cells. The final vehicle concentration was 0.25% to achieve a wide dose range. Caspase-Glo 3/7 reagent (Promega, #G8090) (100 μl) was added to each well and contents were gently mixed using a plate shaker at 300 rpm for 30 s. The plates were incubated at room temperature for 2 h and the luminescence was measured using a Nexcelom Celigo Cytometer.

### Cell Cycle Assay

Cells were seeded at 1 × 10^6^ cells in 10 cm dish and allowed to attach overnight. Compounds were suspended in DMSO, diluted to final concentrations and added to cells for 24 h. The final vehicle concentration was ≤0.25%. Cells were then trypsinized, washed with PBS, and fixed with 70% ethanol for 1 h. After ethanol fixation, cells were washed in PBS and treated with RNAse (100 μg/ml) for 15 min at room temperature. Propidium iodide (100 μg/ml) was added and incubated for 45 min, after which cells were washed with PBS and finally resuspended in 200 μl of PBS. The analysis was done using a Nexcelom K2 Cellometer and histograms were generated for the samples via FCS software.

### Wound Healing Assay for Cell Migration

Cells were seeded at 50,000 cells per well in 24-well plate. Briefly, scratch was made with a 1000 μl filtered tip, medium removed and substituted with serum-free medium containing vehicle or compounds. Pictures were taken immediately following the scratch and 24 h later after treatment, using an AmScope MU900 with Toupview software (AmScope, amscope.com). The percent rate (%) of disclosure was calculated based on the statistics of Image J software.

### Boyden Chamber Assay for Cell Invasion

For invasion assays, 0.5 ml of serum-free medium containing vehicle or compounds was added to each well of a 24-well plate. Matrigel (300 μg/ml) (Sigma, #CLS354234) was added (120 μl) to 8 μm Boyden chamber inserts (Millipore, #PI8P01250) and incubated at 37 °C for ≥1 h. Cells were trypsinized, washed with PBS, and 100,000 cells in serum-free media were seeded per insert. After incubation, cells on the underside of inserts were fixed (4% PFA), permeabilized (70% methanol), and stained with 0.2% crystal violet. Inserts were rinsed and dried overnight. Images were captured using an AmScope MU900 and Toupview software, and invading cells were quantified in ImageJ (NIH) across four fields and normalized to vehicle.

### Live-Dead Staining of *in Vitro* 3D Tumor Spheroids

Cells were seeded at 1000 cells per well in 96 well ultra-low attachment plates and incubated at 37 °C for 14 days, to allow the formation of 3D tumor spheroids. Then, the spheroids were treated with vehicle or compounds for 72 h. Live-dead staining was performed (Thermo Fisher Scientific, #R37601) following manufacturer’s protocol. Images were acquired using a 10 x objective on a Nikon Eclipse E600 microscope using DS-Ri1 digital camera and NIS Elements software (Nikon Instruments, microscope.healthcare.nikon.com/en_AOM/products/software/nis-elements).

### DCFDA assay

the assay was performed using the manufacturer’s protocol (Cayman, #601520).

### Compound Stability in Human Plasma

Plasma stability studies were performed by Pharmaron (Beijing, China). Wheldone (1 mM in DMSO) and propantheline (1 mM in acetonitrile) stock solutions were prepared. Working solutions (4 μl) were spiked into 796 μl human plasma to 5 μM final concentration (0.5% solvent). Aliquots (50 μl) were incubated at 37 °C for 15, 30, 60, or 120 min. Reactions were quenched with 300 μl acetonitrile containing internal standards (500 nM labetalol, 2 μM ketoprofen). Time 0 controls were quenched immediately. Samples were vortexed (5 min) and centrifuged (3220×*g*, 30 min, 4 °C). Supernatants (100 μl) were transferred to a 96-well plate, diluted with 100 μl water, and analyzed by LC-MS/MS to quantify compounds at each time point.

### Metabolic Stability in Human and Mouse Liver Microsomes

Microsomal stability was assessed by Pharmaron (Beijing, China). Reaction mixtures contained 100 mM phosphate buffer, 5 mM MgCl_2_, and 0.5 mg/ml pooled liver microsomes. A 10 mM NADPH solution (40 μl, 1 mM final) was added, or ultra-pure H_2_O for non-enzymatic controls. Mixtures were pre-warmed at 37 °C for 5 min. Reactions began by adding 2 μl of 200 μM test compound or verapamil (1 μM final). Samples were collected at 0, 5, 10, 20, and 60 min. At each time point, 50 μl reaction mixture was quenched with 200 μl ice-cold acetonitrile containing internal standards (200 nM imipramine, 200 nM labetalol, 2 μM ketoprofen). Samples were centrifuged at 3220*g* for 40 min to remove proteins. Supernatants (180 μl) were analyzed by LC-MS/MS to quantify parent compound and assess stability.

### Murine Pharmacokinetics

The mouse pharmacokinetics study was performed at Wuxi AppTec (Shanghai, China). Female C57BL/6 mice (18.6 ± 0.7 g, n = 3 per group) were administered a single dose of wheldone at 1 mg/kg (IV) or 10 mg/kg (IP) in a vehicle of 10% NMP:80% PEG400:10% H_2_O. Blood samples were collected serially into K_2_-EDTA tubes at 0.083, 0.25, 0.5, 1, 2, 4, 8 and 24 h after IV dosing, and at 0.25, 0.5, 1, 2, 4, 8 and 24 h after IP dosing. Plasma samples were collected and stored frozen until determination of plasma wheldone concentrations by LC-MS/MS and subsequent noncompartmental analysis of pharmacokinetic parameters (Phoenix WinNonlin 8.3.5, onlinehelp.certara.com) (17).

### Intraperitoneal Xenograft Study

OVCAR8-RFP cells (5 × 10^6^) were xenografted intraperitoneally into NCr *nu/nu* athymic nude female mice. Tumor growth was monitored using the Lago X System (Spectral Instruments Imaging). Upon detection, mice were treated 3× per week for 3 weeks with taxol (5 mg/kg), wheldone (10 mg/kg), or vehicle (10% NMP:80% PEG400:10% H_2_O). Mice were weighed and imaged weekly (2 s exposure, Fstop 2, 535/620 nm excitation/emission). Abdominal radiant efficiency was quantified with Aura Imaging 4.0 and normalized to day 0. Mice were sacrificed after 3 weeks. All studies were approved by the UIC Animal Care and Use Committee according to AALAC standards.

### DiffPOP

Triplicates of ∼3 million OVCAR3 cells were treated with wheldone (1 μM) or DMSO for 30 min at room temperature. Cells were lysed in 40% Phosphoprotein Buffer A (Takara) supplemented with protease inhibitor cocktail (Thermo Fisher Scientific), using four cycles of sonication (30% amplitude, 55 kHz, 30 s on/15 s on ice). Lysates were cleared by centrifugation (18,000×*g*, 20 min, 4 °C), and protein content was quantified using the Pierce BCA Protein Assay Kit (Thermo Fisher Scientific). Lysates were adjusted to 500 μg in 250 μl DiffPOP lysis buffer (2 μg/μl final). Sequential fractionation was performed using nine increasing concentrations of acidified methanol (90% MeOH/1% acetic acid; final concentrations: 2.62%, 6.45%, 11.98%, 19.80%, 30.53%, 44.53%, 61.38%, 79.03%, and 87.50%). After each addition, samples were vortexed and centrifuged (18,000×*g*, 5 min, 4 °C). Supernatants were removed and pellets stored on ice. All protein pellets were rinsed with cold acetone and air dried. Pellets were resolubilized in 8 M urea/50 mM Hepes (pH 8.5), re-quantified (Pierce BCA Kit), reduced with 5 mM TCEP (Thermo Fisher Scientific), and alkylated with 50 mM chloroacetamide (Sigma). Samples were diluted to 2 M urea with 100 mM Tris (pH 8.5), supplemented with 1 mM CaCl_2_, and digested overnight at 37 °C with trypsin (Promega; 1:50 enzyme-to-protein). Digestion was quenched with 5% formic acid. 1 μg of each peptide fraction was loaded onto Evotips (Evosep) following manufacturer’s instructions and analyzed using an Evosep One LC system in 30 Samples Per Day mode with a 44-min gradient. Peptides were separated on an EV1106 C18 silica column (Evosep) and eluted into a Q Exactive HF Orbitrap Mass Spectrometer (Thermo Fisher Scientific). LC solvents included 0.1% formic acid in H_2_O (Buffer A) and 0.1% formic acid in ACN (Buffer B). DIA acquisition was performed in positive ion mode, with MS1 resolution set to 45,000, AGC target 5e6, isolation window of 25 m/z, first mass 300 m/z, and stepped NCE at 30. Raw data were processed using FragPipe v22.0 (DIA_SpecLib_Quant workflow, https://github.com/Nesvilab/FragPipe/releases). No external spectral library was provided. Peptide identifications were performed by searching acquired spectra against the UniProt human reference proteome (UP000005640, version 2022-06-02; 158,202 total entries including 50% reversed decoys). Spectral information was derived internally from the DIA data to build a project-specific library using MSFragger-DIA and EasyPQP, followed by precursor quantification using DIA-NN. Precursor and fragment mass tolerances were set to ±20 ppm. Trypsin digestion was set with up to 2 missed cleavages. Fixed modification: carbamidomethylation (CAA, Sigma) of cysteine. Variable modifications included methionine oxidation and N-terminal acetylation. FDR was set to 1% at both peptide and protein levels. Peptide lengths were restricted to 7 to 50 amino acids and mass range 500 to 5000 Da. MS1 quantification used Match Between Runs.

### Kinesin ATPase Endpoint Assay

The assay was performed using the manufacturer’s protocol (Cytoskeleton, #BK053).

### Global Proteomics (DIA Proteomics)

Cells 3 biological and 2 technical injections (OVCAR3, OVCAR8, PEO1, and OV81.2), washed with PBS, scrapped, and centrifuged. Cell pellets were resuspended in PBS, proteins were extracted via sonication, and protein quantification was performed via the Bradford assay (Bio-Rad). LC-MS/MS sample (20 μg in 15 μl) preparation was performed as described previously (18). Peptides were resuspended in water with 0.1% formic acid (FA) and analyzed using nanoElute 2 coupled to a TimsTOF HT mass spectrometer (Bruker Daltonics). The chromatography column consisted of a 25 cm long, 150 μm i.d. microcapillary packed with 1.5 μm C18 particles (Bruker Daltonics/Pepsep) and was capped by a 20 μm emitter (Bruker Daltonics). LC solvents were 0.1% FA in H2O (Buffer A) and 0.1% FA in MeCN (Buffer B). Peptides were eluted into the MS at a flow rate of 500 nl/min over a 60 min linear-gradient (5–35% Buffer B) at 50 °C. Data was acquired via dia-PASEF using 20 MS/MS windows (optimized using py_DiAID) per one MS1 scan (19). The method covered m/z 400 to 1200 and scan range between 0.6 to 1.4 1/K0. Ramp time was set to 100 ms and total cycle lasted 2.23 s. Collision energy was set as a linear increase from 20 eV at 1/K0 = 0.6 to 59 eV at 1/K0 = 1.6. The MS data was analyzed with DIA-NN (V1.8.1), a generated human peptide library, and the Uniprot human proteome with contaminants as database. The precursor FDR was set to 1% and Match Between Runs was checked. Log2 fold change and −Log10 *p*-values were calculated merging all the 3 biological and 2 technical injections per condition together. As the data is DIA, 1 peptide per protein was standard and no intensity thresholds were applied. Proteins to be kept needed to be quantified in at least 2 out of 6 injections per condition to obtain a *p*-value.

### Mass Spectrometry TMT Labeling Proteomics

For TMT analysis of OVCAR3 lysate, three biological replicates per condition (wheldone 1 μM or DMSO) were labeled with TMT6plex and analyzed on a Q Exactive HF with 90-min LC gradients. Cells were lysed in TNI buffer (50 mM Tris pH 7.5, 250 mM NaCl, 0.5% Igepal CA-630, 1 mM EDTA) with protease inhibitors. Proteins were methanol/chloroform precipitated, resolubilized in 8 M urea/100 mM Tris pH 8.5, reduced (5 mM TCEP), alkylated (50 mM CAA), and digested overnight with trypsin (1:50). Digestion was quenched with 5% formic acid. TMTsixplex labeling (Thermo Fisher Scientific) was performed per manufacturer’s instructions (20). Reporter ions were assigned as: 126.1 (DMSO N1), 127.1 (wheldone N1), 128.1 (DMSO N2), 129.1 (wheldone N2), 130.1 (DMSO N3), 131.1 (wheldone N3). Samples were analyzed on a Q-Exactive HF Orbitrap coupled to a Dionex Ultimate 3000 and separated using a 90-min gradient on a C18 RSLC column. MS1 and MS2 resolutions were 120,000 and 60,000 with NCE 32 and scan range 375 to 1400 m/z. Data were processed in FragPipe v 17.1 against the UniProt human database (UP000005640, version 2020-06-13; 149,622 total entries including 50% reversed decoys) with trypsin specificity (2 missed cleavages), fixed modifications (CAA alkylation, TMT on N-termini), and variable modifications (oxidized Met, N-terminal acetylation, TMT on Lys). Precursor and fragment mass tolerances were set to ±20 ppm. FDR was set to 1%. TMT quantification used TMT-Integrator with default 6-plex settings, followed by median centering and variance scaling.

Data were processed in FragPipe v17.1 against the UniProt human proteome; reporter ions were quantified using TMT-Integrator with median centering and variance scaling. Statistical analysis: differential protein abundance was assessed via a volcano plot using raw *p*-values threshold at 0.05 and log2 fold-change cutoff ±0.38 (∼30% change); Technical reproducibility, data preparation: missing reporter ion values were replaced with the lowest observed intensity for that protein within the replicate set, ensuring all proteins could be included in analysis.

### Data Analysis and Quantification for DiffPOP

For DiffPOP analysis of OVCAR3 lysate, three biological replicates per condition (wheldone 1 μM or DMSO) were fractionated into nine sequential methanol fractions and analyzed by DIA on a Q Exactive HF Orbitrap (Evosep 30 Samples Per Day, 44 min gradient). MS1 resolution 45,000, AGC 5E6, Max IT 200 ms; MS2 resolution 30,000, 30 overlapping windows, isolation 25 m/z, stepped NCE 30; total cycle time ∼1.7 s. Data were processed with FragPipe v22.0 (library-free mode, UniProt human proteome). Fractions and replicates were processed in randomized order to minimize batch effects. No internal reference standards were used for RT calibration; missing reporter intensities were aligned via retention time and filled using Skyline prior to statistical testing. Shifts in protein solubility profiles were then identified using the Kolmogorov–Smirnov test across fractions. Due to the small number of fractions (n = 9), the top 3% of Kolmogorov–Smirnov scores were considered significant. Technical reproducibility was assessed via Spearman correlations >0.7 across replicates. Analysis and protein-level quantification was derived from peptide intensities across nine DiffPOP fractions using the FragPipe DIA_SpecLib_Quant workflow. Only proteins identified at 1% FDR were considered. For TMT6plex analysis, TMT reporter ion intensities were extracted and quantified using TMT-Integrator with default six-plex settings. Protein-level quantification was derived from summed peptide reporter intensities following median centering and variance scaling. Only proteins passing 1% FDR were included in downstream analysis; no additional minimum peptide count threshold was imposed beyond FragPipe defaults. Missing reporter ion values were replaced with the lowest observed intensity for the corresponding protein within each replicate set to permit log2 fold-change calculation and volcano plot analysis. No explicit outlier rejection was applied. Differential protein abundance was assessed using raw *p*-values (*p* < 0.05) and a log2 fold-change threshold of ±0.38 (∼30% change). Technical reproducibility was evaluated at the biological replicate level through consistency of reporter ion measurements following normalization.

### Experimental Design and Statistical Rationale

Data presented are the mean ± SEMand represent at least 3 independent experiments. Statistical analysis was carried out using GraphPad Prism software and specified in the figure legends for each experiment. Symbols used to signify significance include: ∗ denotes *p* < 0.05.

## Results

### Wheldone is Selectively Cytotoxic to HGSOC Cells

**Fig. 1:**
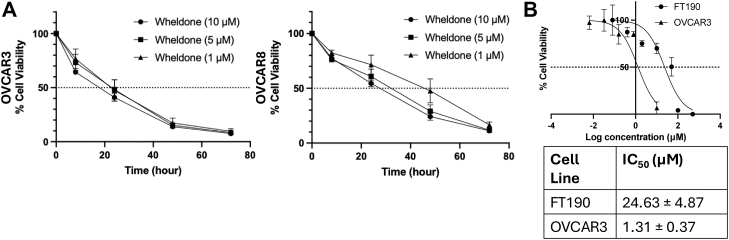
**Wheldone is selectively cytotoxic to HGSOC cells.***A*, OVCAR3 and OVCAR8 cells were treated with wheldone at three concentrations (1 μM, 5 μM, 10 μM). Cell viability was measured at 0, 8, 24, 48, and 72 h. *B*, FT190 or OVCAR3 cells were plated and treated with vehicle (DMSO) or wheldone for 72 h. IC_50_ value was generated from the dose-response curve.

Wheldone, which was isolated from a fungal-fungal coculture (5), was reported to display cytotoxicity in MDA-MB-231, MDA-MB-435, and OVCAR3 cancer cells (6). To further focus on HGSOC, OVCAR3 and OVCAR8 were tested at three concentrations of wheldone at different time points (Fig. 1*A*). Next, wheldone was evaluated in a non-tumorigenic fallopian tube secretory epithelial cell line, FT190 (Fig. 1*B*) (21). Wheldone had an IC_50_ of 24.63 μM in FT190 cells, which was significantly higher than that observed in the OVCAR3 (reported as 1.31 μM). This difference in cytotoxicity indicates that wheldone has a degree of selectivity, showing greater potency against tumorigenic cells while being less toxic to non-tumorigenic cells.

### Wheldone Leads to Apoptosis in a Caspase 3/7-Dependent Manner

**Fig. 2:**
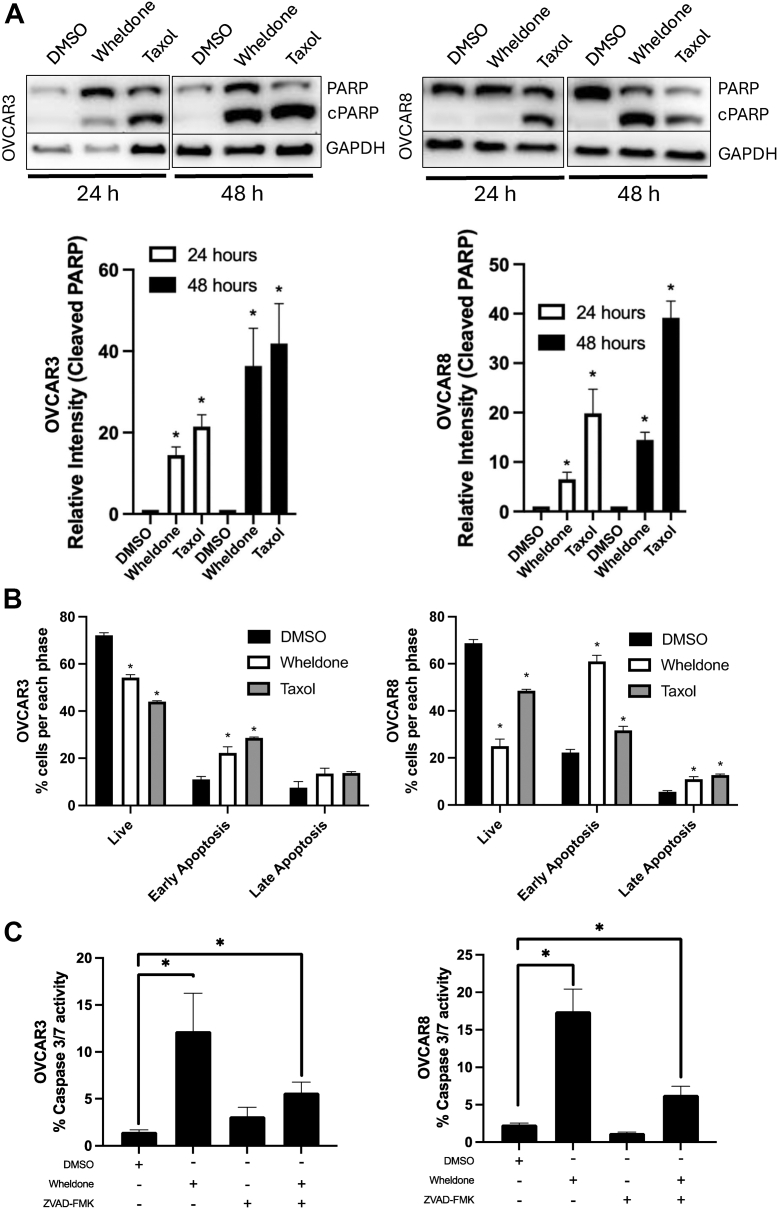
**Wheldone induces caspase-dependent apoptosis.***A*, OVCAR3 and OVCAR8 cells, treated with vehicle (DMSO), wheldone (1 μM), or taxol (50 nM) for 24 or 48 h. Representative western blots show an increase in the apoptosis marker, cleaved PARP. GAPDH serves as a loading control. Densitometry for three blots shows average relative intensity. *B*, OVCAR3 and OVCAR8 cells were treated with vehicle (DMSO), wheldone (1 μM), or taxol (50 nM) for 24 h, and stained with annexin V-FITC (AV) and propidium iodide. Fluorescent signal (n = <2000 cells per replicate) was quantified with a Nexcelom Cellometer. Percentages of non-apoptotic cells, early and late apoptotic cells were quantified. *C*, OVCAR3 and OVCAR8 cells were treated with vehicle (DMSO), wheldone (1 μM), or ZVAD-FMK (10 μM) for 24 h and stained with Caspase-Glo 3/7 reagent. Luminescence signal was quantified with a Nexcelom Celigo Cytometer. Percentages of the caspase 3/7 activity were quantified. Each experiment was performed in three biological replicates and data represent triplicate values ± SEM. For *panels A* and *B*, one-way ANOVA with Dunnett’s multiple comparisons was used to compare to the vehicle and to generate *p* values (∗ denotes *p* < 0.05). For *panel C*, student’s *t* test was used to compare to the vehicle and to generate *p* values (∗ denotes *p* < 0.05).

To investigate the mechanism of cell death, western blotting showed increased cleaved PARP after wheldone treatment in OVCAR3 (24 h) and OVCAR8 (48 h) (*p* < 0.05) (Fig. 2*A*). Apoptosis was confirmed by Annexin V–FITC/Propidium Iodide staining, with significant increases in early apoptosis after 24 h after wheldone treatment in both OVCAR3 and OVCAR8 cell lines (Fig. 2*B*). A caspase 3/7 reporter assay showed that wheldone-induced apoptosis was caspase-dependent, as activity was elevated with treatment and blocked by the pan-caspase inhibitor ZVAD-FMK in both OVCAR3 and OVCAR8 cell lines (*p* < 0.05) (Fig. 2*C*).

### Wheldone Decreases Migration, Invasion, *in Vitro* 3D tumor Spheroid Size and Reduces 2D foci

**Fig. 3:**
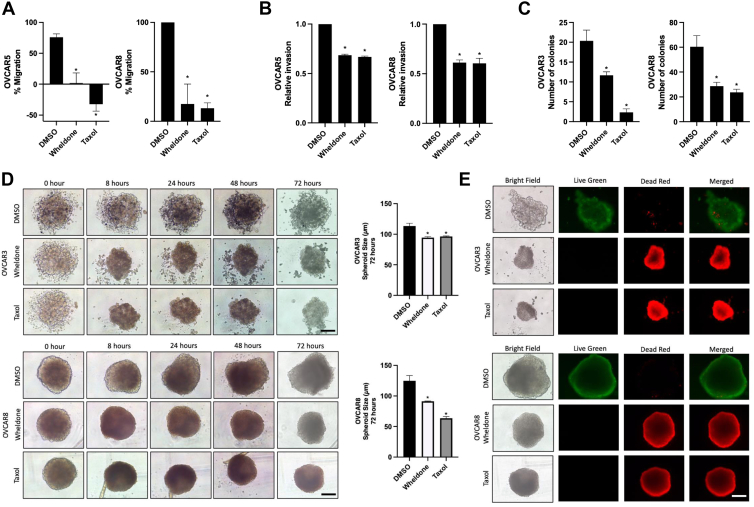
**Wheldone decreases migration and invasion, increases cell death in *in vitro* 3D tumor spheroids, and leads to cell death in a cytostatic manner.***A*, OVCAR5 and OVCAR8 cells were treated with vehicle (DMSO), wheldone (1 μM), or taxol (50 nM) for 24 h, immediately following the scratch. Percentages of closure of the scratch (wound) were quantified. *B*, OVCAR5 and OVCAR8 cells were treated with vehicle (DMSO), wheldone (1 μM), or taxol (50 nM) for 24 h. Percentages of permeabilization through the Boyden chamber with matrigel were quantified. *C*, OVCAR3 and OVCAR8 cells were treated with vehicle (DMSO), wheldone (1 μM), or taxol (50 nM) for 72 h. Foci were then allowed to grow in non-treated medium for 20 days. Foci counts were then quantified and normalized to the vehicle. *D*, *In vitro* 3D cancer cell spheroids (OVCAR3, OVCAR8) were allowed to form 14 days and treated with vehicle (DMSO), wheldone (1 μM), or taxol (50 nM) for 72 h. Diameters of spheroids were measured at 72 h. *E*, spheroids were then stained with Live-Dead stain (dead = EtBr red, live = calcein green). Immunofluorescence images were taken at 10 X magnification. Scale bar is at 500 μm. Each experiment was performed in three biological replicates and data represent triplicate values ± SEM. Student’s *t* test was used to compare to the vehicle to wheldone *p* values (∗ denotes *p* < 0.05).

Wound healing and invasion assays showed that wheldone significantly reduced migration and invasion in OVCAR5 and OVCAR8 (*p* < 0.05) (Fig. 3, *A* and *B*), while OVCAR3 was excluded due to poor motility *in vitro*. A 2D foci assay demonstrated decreased colony formation in OVCAR3 and OVCAR8, suggesting cytostatic effects (Fig. 3*C*). In 3D spheroid models, wheldone reduced spheroid size and increased cell death in OVCAR3 and OVCAR8 as indicated by loss of calcein green and elevated propidium iodide staining (*p* < 0.05) (Fig. 3, *D* and *E*).

### Wheldone did Not Reduce tumor Burden *in Vivo*

**Fig. 4:**
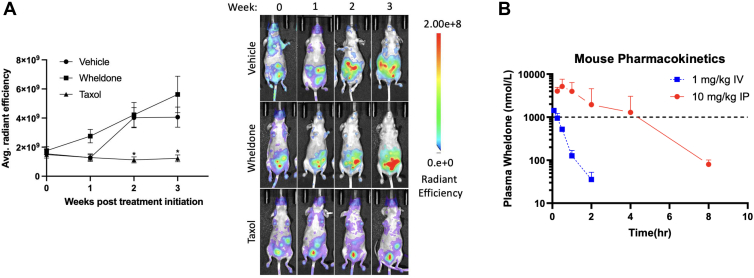
**Wheldone failed to reduce tumor burden *in vivo* due to rapid clearance.***A*, OVCAR8-RFP cells were xenografted intraperitoneally and allowed to form tumors. Mice were dosed with vehicle (10% NMP:80% PEG400:10% H_2_O) (n = 2), taxol (5 mg/kg) (n = 2), or wheldone (10 mg/kg) (n = 4) 3 times per week for 3 weeks. Quantification of tumor burden over time (average radiant efficiency as measured with Lago X System) was normalized to day 0, and representative images of OVCAR8-RFP tumors in mice are displayed. *B*, female C57BL/6 mice (n = 3/group) were given either 1 mg/kg IV or 10 mg/kg IP and plasma assessed for wheldone levels at various time points for 24 h following dosing. Later timepoints having less than three animals exhibiting wheldone levels above the lower limit of quantification (LLOQ, 23.2 nM) were excluded from the plot. Horizontal reference line at 1 μM represents likely therapeutic levels of wheldone. Plotted data represent means of triplicate values ± SD. C_0_ = concentration at time 0 (IV only), C_max_ = maximum concentration (IP only), T_max_ = time at which C_max_ occurs (IP only), AUC_last_ = Area under the curve until the last measurable plasma concentration, CL = systemic clearance (calculated for IV only), T_1/2_ = elimination half-life, VD_ss_ = volume of distribution at steady state (IV only). Each experiment was performed in three biological replicates and data represent triplicate values ± SEM. Student’s *t* test or one-way ANOVA with Dunnett’s multiple comparisons was used to generate *p* values (∗ denotes *p* < 0.05).

To evaluate the ability of wheldone to reduce OVCAR8-RFP xenografts, female mice were injected with OVCAR8-RFP cells IP and monitored for tumor formation (Fig. 4*A*). Single doses exceeding 10 mg/kg for wheldone caused body weight loss, and thus, studies were performed at 10 mg/kg. Upon establishment of tumors, the mice were dosed IP with wheldone, taxol or vehicle, three times per week for 3 weeks. Wheldone did not decrease the tumor burden based on average abdominal radiant efficiency as compared with vehicle at week 3, while taxol achieved significant tumor burden reduction by week 2. Wheldone did not exhibit toxicity based on weight loss in mice.

### Wheldone is Metabolically Stable *in Vitro* but Rapidly Cleared *in Vivo*

To assess whether wheldone’s limited *in vivo* efficacy was due to metabolism, stability was evaluated in human plasma and liver microsomes. In plasma, wheldone showed high stability (T_1/2_ > 511 min; >92% remaining at 120 min), comparable to propantheline (Supplemental Table S4). In pooled human and mouse liver microsomes, wheldone was also highly stable (T_1/2_ > 255 min; CL_int_ < 5.42 μl/min/mg), with no significant NADPH-dependent metabolism (Supplemental Tables S5 and S6), indicating metabolic instability is not a concern. Pharmacokinetic studies in mice showed rapid clearance after 1 mg/kg IV dosing (CL = 50 ml/min/kg; T_1/2_ = 0.8 h), and a large volume of distribution (2.4 L/kg), suggesting strong tissue distribution. After 10 mg/kg IP dosing, absorption was complete (100% bioavailability), but plasma concentrations >1 μM were maintained for only ∼4 h (Fig. 4*B*, Supplemental Table S7), suggesting short exposure duration, not metabolic degradation, may limit *in vivo* activity.

### Wheldone Targets KIF11, an Essential Cell Cycle Protein in HGSOC

**Fig. 5:**
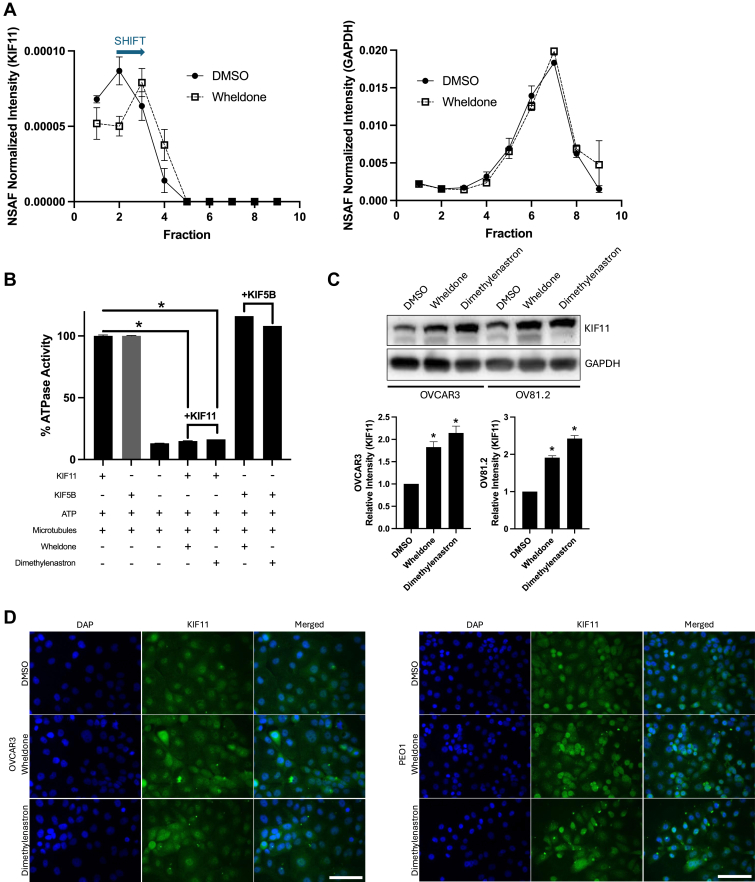
**Wheldone targets KIF11.***A*, differential precipitation of proteins was performed in OVCAR3 cells. Normalized spectral abundance factor- intensity of KIF11 across fractions 1 to 10 was compared in vehicle (DMSO)-treated and wheldone-treated cells, using GAPDH as the loading control. KIF11 levels for wheldone-treated cells peak at fraction 3, while KIF11 levels for DMSO-treated cells peak at fraction 2, demonstrating the shift in protein precipitation. *B*, ATPase activity assay showing inhibition of KIF11 (reduced ATPase activity) but not KIF5B in cells treated with wheldone (1 μM) and dimethylenastron (200 nM). *C*, OVCAR3 and OV81.2 cells were treated with vehicle (DMSO), wheldone (1 μM), or dimethylenastron (200 nM) for 24 h. Representative western blots show an increase in KIF11. GAPDH serves as a loading control. *D*, cells (OVCAR3, PEO1) were treated with vehicle (DMSO), wheldone (1 μM), or dimethylenastron (200 nM) for 4 h. Cells were then stained with KIF11 and DAPI. Immunofluorescence images were taken at 40X magnification. Scale bar is at 20 μm. Each experiment was performed in three biological replicates and data represent triplicate values ± SEM. One-way ANOVA with Dunnett’s multiple comparisons was used to compare to the vehicle and to generate *p* values (∗ denotes *p* < 0.05).

Because wheldone demonstrated significant promise *in vitro*, but failed to demonstrate efficacy *in vivo*, we need to identify the protein target in order to make plan for potential future structural modifications to improve *in vivo* efficacy through either modulating clearance or target affinity. To identify direct target proteins of wheldone, DiffPOP, was performed (16). By using varying concentrations of methanol or ethanol to induce protein precipitation into roughly 10 fractions, DiffPOP provides fine-tuned control over the denaturation process, offering better reproducibility compared to temperature ramping or enzymatic digestion. Furthermore, the assay is conducted within a single sample, reducing the number of required samples and conserving precious natural product lead compounds. Wheldone binding was tested for its ability to stabilize target proteins, causing delayed precipitation in response to an acidified methanol gradient. This shift reflects increased unfolding resistance. A total of 299 proteins showed altered precipitation, with 18 classified as high-interest targets based on significant shifts (*p* < 0.05) and elution at higher methanol concentrations than vehicle (Fig. 5*A*, Supplemental Fig. S1). KIF11 exhibited the most pronounced shift, while GAPDH, used as a control, showed no change.

A KIF11 ATPase assay was used to assess whether wheldone binding also inhibited enzymatic activity. Decreased ATPase activity for KIF11 means that the protein is being inhibited by the compound of interest (22). Both wheldone and a known KIF11 inhibitor, dimethylenastron, reduced ATPase activity, while no effect was seen with the negative control kinesin, KIF5B (Fig. 5*B*). KIF11 expression in HGSOC cell lines was confirmed by Western blot (Supplemental Fig. S2), and levels increased after wheldone treatment (Fig. 5*C*). Immunofluorescence revealed puncta formation following treatment with wheldone or dimethylenastron, suggesting similar inhibitory effects and altered KIF11 localization (Fig. 5*D*).

### Proteomics Revealed Expression of Proteins Impacted by Wheldone Treatment

**Fig. 6:**
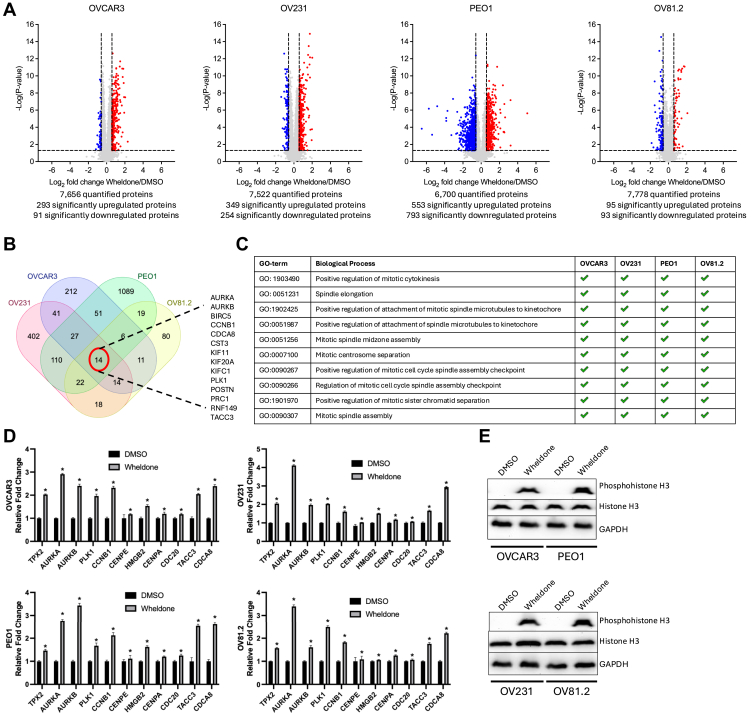
**Downstream regulations in mitosis and cell cycle further indicate that wheldone inhibits KIF11.***A*, OVCAR3, OV231, PEO1, and OV81.2 cells were treated with vehicle (DMSO) or wheldone (1 μM) for 24 h. Global proteomics profiling was performed and compared with the vehicle. The red dots represent significantly upregulated proteins, and blue dots are significantly downregulated proteins. *B*, Significantly regulated proteins from the 4 cell lines were overlapped on the Venn diagram to show that there is an alignment of 14 regulated proteins across the 4 cell lines treated with wheldone. *C*, gene Ontology (GO) analysis was performed on all the significantly regulated proteins. *D*, several proteins associated with KIF11 inhibition from other known inhibitors were regulated in all 4 cell lines. *E*, whole cell lysates from OVCAR3, OV231, PEO1, and OV81.2 cells, treated with vehicle (DMSO) or wheldone (1 μM) for 24 h. Representative western blots show an increase in the mitotic arrest marker, phosphohistone H3; GAPDH serves as a loading control. Each experiment was performed in three biological replicates and data represent triplicate values ± SEM. Student’s *t* test was used to generate *p* values (∗ denotes *p* < 0.05).

Global DIA proteomics (Fig. 6*A*) revealed signaling changes consistent with KIF11 inhibition. We identified 14 proteins, including mitotic regulators KIF11, AURKA, AURKB, BIRC5, and PLK1, were consistently altered across all cell lines (Fig. 6*B*), indicating disruption of spindle assembly and cytokinesis, consistent with known KIF11 inhibitors (23). Gene Ontology analysis confirmed enrichment in mitotic processes such as “spindle elongation” and “mitotic spindle assembly” (Fig. 6*C*), supporting a mechanism aligned with known KIF11 inhibitors. Proteomics analysis showed that wheldone upregulated 11 proteins typically induced by KIF11 inhibition, TPX2, aurora kinases, PLK1, CCNB1, CENPA, CENPE, HMGB2, CDC20, CDCA8, and TACC3 (11, 23, 24, 25), across 4 cell lines (*p* < 0.05) (Fig. 6*D*), supporting mitotic arrest. Western blot confirmed increased phospho-histone H3, a marker of mitotic catastrophe (*p* < 0.05) (Fig. 5*E*) (26), further suggesting wheldone acts as a KIF11 inhibitor.

### Wheldone Leads to G2/M Arrest and Changes in Microtubule Structures

**Fig. 7:**
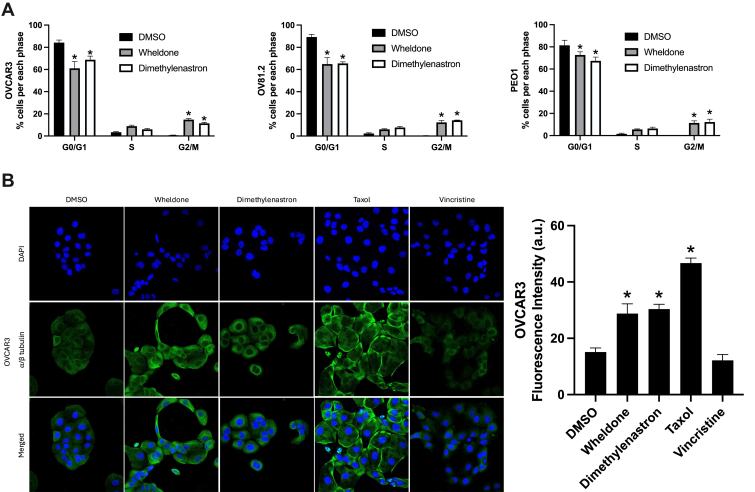
**Wheldone induces G2/M arrest with structural changes in microtubules.***A*, OVCAR3, OV81.2 and PEO1 cells were treated with vehicle (DMSO), wheldone (1 μM), or dimethylenastron (200 nM) for 24 h and stained with propidium iodide. Cell cycle phases were analyzed with a Nexcelom Cellometer and percentages were quantified. *B*, OVCAR3 and PEO1 cells were treated with vehicle (DMSO), wheldone (1 μM), dimethylenastron (200 nM), taxol (50 nM), or vincristine (50 nM) for 4 h. Cells were then stained with anti-*α*/β tubulin antibodies followed by fluorescently labeled secondary antibody and DAPI. Immunofluorescence images were taken at 40 X magnification. The scale bar is at 20 μm. Fluorescence intensity was quantified and compared to the vehicle.

KIF11 inhibition causes G2/M arrest; therefore a cell cycle analysis was performed to confirm if this occurs with wheldone treatment. Wheldone treatment led to significant G2/M cell cycle arrest in OVCAR3, OV81.2, and PEO1 cells (*p* < 0.05) (Fig. 7*A*). Next, wheldone’s effects on microtubule structures were assessed via immunofluorescence staining, using dimethylenastron, a microtubule stabilizer, taxol, and a microtubule destabilizer, vincristine as controls (Fig. 7*B*). Dimethylenastron, a known KIF11 inhibitor, treatment results in structural changes in microtubules in OVCAR3 cells that resemble those from treatment with wheldone.

### Wheldone Downregulates HNRNPD, a DNA Damage Repair Protein

TMT-labeled proteomics in OVCAR3 cells identified HNRNPD, a DNA damage repair protein (27), as one of the most significantly downregulated targets following wheldone treatment (Supplemental Fig. S3*A*). Given the clinical success of PARP inhibitors in BRCA1/2-mutant or epigenetically silenced HGSOC with homologous recombination deficiency, we investigated whether wheldone-induced HNRNPD downregulation could enhance PARP inhibitor efficacy via synthetic lethality (27, 28). HNRNPD suppression was confirmed by Western blot in four HGSOC cell lines (*p* < 0.05) (Supplemental Fig. S3*B*). Increased γH2AX in OVCAR3 and OVCAR8 indicated DNA damage (Supplemental Fig. S3*C*), which was attenuated by co-treatment with N-acetyl-L-cysteine (NAC), along with reduced PARP cleavage and early apoptosis (Supplemental Fig. S3, *D* and *E*), implicating ROS in cytotoxicity. Pyocyanin was used as the positive control for producing ROS (29). Wheldone elevated ROS in chemoresistant lines PEO4, isolated post-cisplatin resistance from PEO1 (30), and OV231 CP30, derived from OV231 after 30 passages of cisplatin exposures (31), and NAC reversed this effect (Supplemental Fig. S3*F*), suggesting ROS, not HNRNPD loss, drives apoptosis. Although OV81.2 CP40 (HNRNPD-null) was resistant, wheldone remained effective in both HNRNPD-low OV231 and HNRNPD-high OV231 CP30 cells, indicating HNRNPD is not the primary target (Supplemental Fig. S4). Collectively, these results support ROS-mediated apoptosis as the dominant mechanism of wheldone cytotoxicity.

### Combination of Wheldone with AKR1C3 or CDK4/6 Leads to Cell Death in OV81.2 CP40

Wheldone was ineffective in OV81.2 CP40, a platinum-resistant derivative of OV81.2, despite retaining cytotoxicity in the parental OV81.2 (Supplemental Table S2). Proteomics analysis (Supplemental Fig. S4*A*) revealed increased AKR1C3 and CDK6 expression in OV81.2 CP40 cells, with Gene Ontology term enrichment confirming continued effects on mitosis and cell cycle pathways in both lines (Supplemental Fig. S4*B*). Proteomics validated significantly higher AKR1C3 and CDK6 levels in CP40 after wheldone treatment (Supplemental Fig. S4*C*). Co-treatment with abemaciclib, CDK4/6 inhibitor, or ASP9521, AKR1C3 inhibitor, at concentrations optimized in Supplemental Table S3, enhanced wheldone’s cytotoxicity (Supplemental Fig. S4*D*). These data suggest that AKR1C3 and CDK6 upregulation contributes to resistance and that targeting these pathways may restore sensitivity to wheldone in chemoresistant HGSOC. These combinations may also hold promise for increasing *in vivo* activity of wheldone.

## Discussion

Wheldone, a novel fungal metabolite, shows potential as a cytotoxic agent in HGSOC by inhibiting KIF11. This aligns with existing KIF11 inhibitor literature but offers a unique chemical structure to overcome limitations. Structurally, wheldone features an unprecedented scaffold distinct from the well-characterized dihydropyrimidine and quinazolinone-based KIF11 inhibitors (9, 23), providing a new chemical space for modulating mitotic kinesins. DiffPOP profiling and ATPase assays validated KIF11 interaction. Importantly, the DiffPOP method confirmed that wheldone directly engages KIF11 through conformational population analysis. DiffPOP profiling and ATPase assays validated KIF11 interaction. Wheldone treatment (or dimethylenastron) caused G2/M cell cycle arrest, suggesting KIF11 inhibition disrupts mitosis, consistent across multiple cell lines. Upregulation of TPX2, AURKA, AURKB, and PLK1, and increased phosphohistone H3, further support mitotic arrest and overlap with downstream effects of existing KIF11 inhibitors (23). Apoptosis is likely caspase 3/7 dependent.

Beyond its cytotoxic profile, wheldone exhibits structural novelty compared to other KIF11 inhibitors. Wheldone also disrupted hallmarks of cancer, including cell migration, invasion, and *in vitro* spheroid growth. It demonstrated selective cytotoxicity, with an IC_50_ selectivity index of nearly 20-fold (18.8) in OVCAR3 over non-cancerous FT190, indicating high selectivity and potential for further development, possibly via analog synthesis (32). Proteomics identified HNRNPD downregulation, which plays a role in DNA repair (27). This suggests potential for synthetic lethality in combination with PARP inhibitors for homologous recombination-intact HGSOC patients, where PARP inhibitors alone are ineffective (33). However, wheldone retained cytotoxicity in HNRNPD-expressing OV231 cells, supporting KIF11 inhibition as its primary cytotoxic target beyond HNRNPD modulation.

Despite clinical trial challenges for other KIF11 inhibitors (lack of efficacy/off-target effects) (34), high KIF11 expression correlates with poor patient survival (9), underscoring its therapeutic potential (35). Wheldone treatment at 10 mg/kg caused no significant weight loss, supporting *in vitro* findings. A murine pharmacokinetic study revealed rapid systemic clearance, resulting in minimal time at therapeutic levels, despite excellent *in vitro* metabolic stability in liver microsomes. This discrepancy highlights the need for further metabolism studies in more complex systems like primary hepatocytes, as microsomal preparations may over-predict stability for compounds metabolized by non-CYP450 pathways (36). The lower potency and rapid clearance likely explain the lack of *in vivo* tumor reduction. These studies also highlight that now that the target for wheldone has been identified, structural modifications based on molecular interactions could be rationally designed to ensure binding and reducing drug clearance.

Target identification is a decisive step in advancing phenotypic hits into drug candidates, particularly for complex natural products where polypharmacology is common. Proteomic target-deconvolution approaches enable unbiased, proteome-wide assessment of compound engagement in a native cellular context, revealing both primary targets and off-target liabilities while simultaneously reporting pathway-level consequences. Among these methods, DiffPOP provides a simple, sample-sparing way to convert ligand-induced stability changes into fractionation shifts, improving reproducibility and throughput relative to thermal or enzymatic perturbations and facilitating orthogonal validation cascades. In this study, DiffPOP prioritized KIF11 as a high-confidence interactor and, together with biochemical ATPase inhibition, G2/M arrest, mitotic biomarker modulation, and global proteomic signatures of spindle assembly disruption, established on-target activity. Defining KIF11 as the molecular target now enables structure-guided optimization (e.g., improving affinity and residence time), rational selection of pharmacodynamic biomarkers (such as phospho-histone H3, TPX2, and AURKA), and de-risking of translational strategies including ADC payload deployment by clarifying therapeutic index and off-target risks. More broadly, integrating target-ID proteomics with global quantitative proteomics accelerates structure activity relationships, informs combination strategies to overcome resistance, and provides the mechanistic confidence needed for preclinical development (19).

Overall, wheldone's anti-mitotic effects, minimal non-cancerous cell toxicity, and rapid clearance support its further development possibly by improving the potency through chemical modifications informed by structural biology. Computational approaches should be used to guide structural changes that may increase potency. If the compound could be improved it might be used as a cytotoxic payload for antibody-drug conjugates (ADCs), a successful strategy for natural product anticancer agents (37). Equally, its confirmed target engagement with KIF11 makes it an informative inhibitor to explore mitotic dependency and resistance mechanisms in HGSOC. Identification of the molecular target is a key step for developing strategies to link a molecule to an ADC because they inform which cell types will respond to the payload, which portions of the molecule are required for activity, and whether the target would allow for adjacent cells in the microenvironment to also be eliminated through the bystander effect. This study identifies wheldone as a novel KIF11 inhibitor and highlights its ability to kill chemoresistant cells such as PEO4 and OV231. Quantitative data implicated elevated AKR1C3 and CDK6 in OV81.2 CP40 cells with wheldone resistance. Future efforts will focus on optimizing wheldone through ADC delivery, chemical modification, and computational modeling of KIF11 binding.

## Data Availability

All raw and processed mass spectrometry proteomics data generated in this study, including DiffPOP DIA datasets, global DDA-PASEF proteomes, and TMT-labeled experiments, together with search results and spectral libraries, have been deposited to the ProteomeXchange Consortium via the PRIDE accession PXD070302. Downstream global proteomics is under accession PXD070302 token ZjkADT3QVcDR. The submissions include raw instrument files, metadata, FragPipe parameter files, peptide/protein identification tables, and quantification matrices. Reviewer access credentials will be provided to the journal during peer review, and all datasets will be made public upon publication.

## Supplemental data

This article contains supplemental data.

## Conflict of Interest

The authors declare no competing interests.
