# Supplementary material for: Natural Product Target Identification of Wheldone, a Fungal Metabolite, as a KIF11 Inhibitor in Ovarian Cancer Using the DiffPOP (Differential Protein Precipitation) Method
Source: Mol Cell Proteomics. 2026 Mar 23;25(5):101558. doi: 10.1016/j.mcpro.2026.101558 (PMC13122309; doi:10.1016/j.mcpro.2026.101558)
Supplement: Supplementary Figures and Tables [file mmc1.docx]

**Supplementary Information**


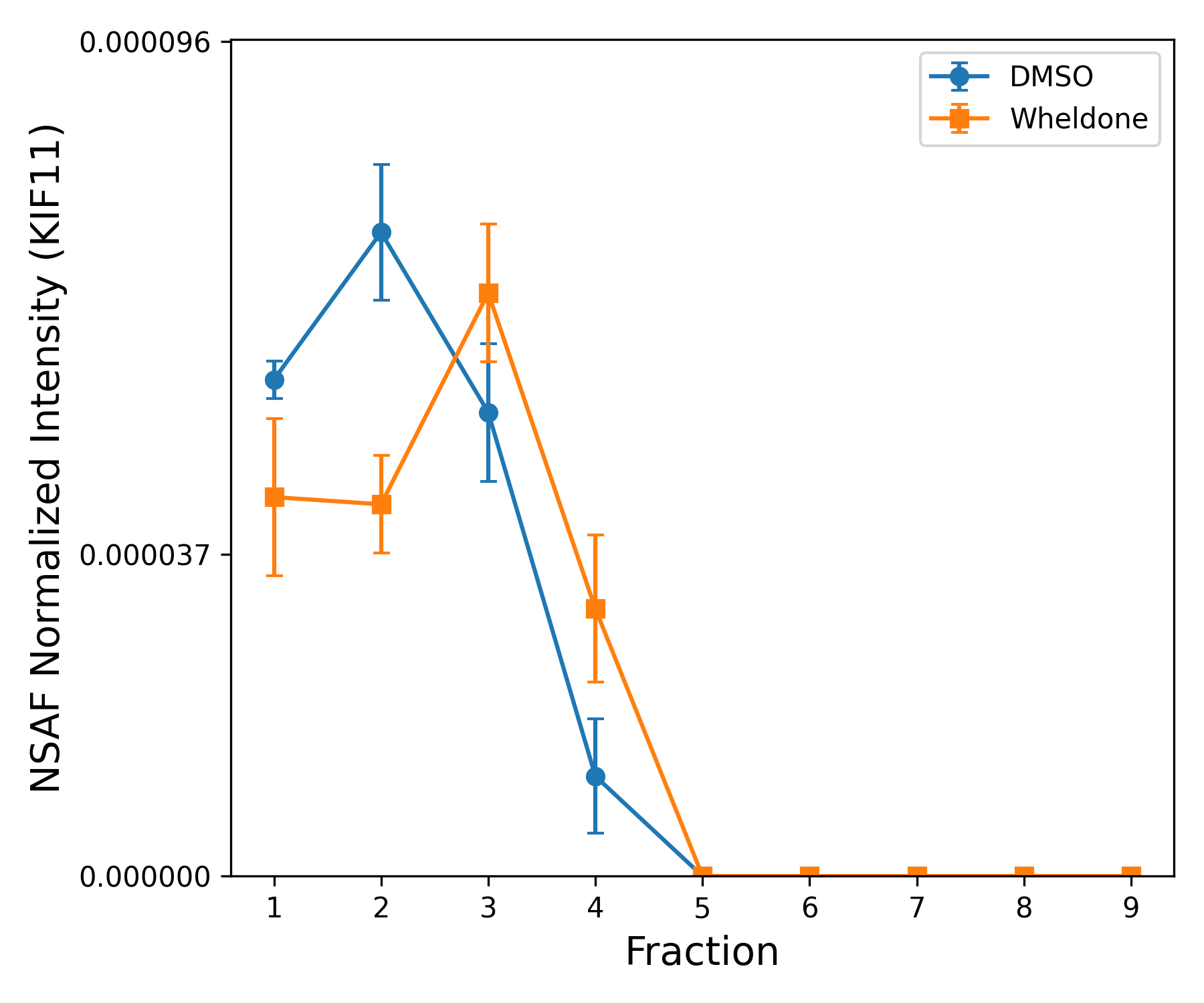

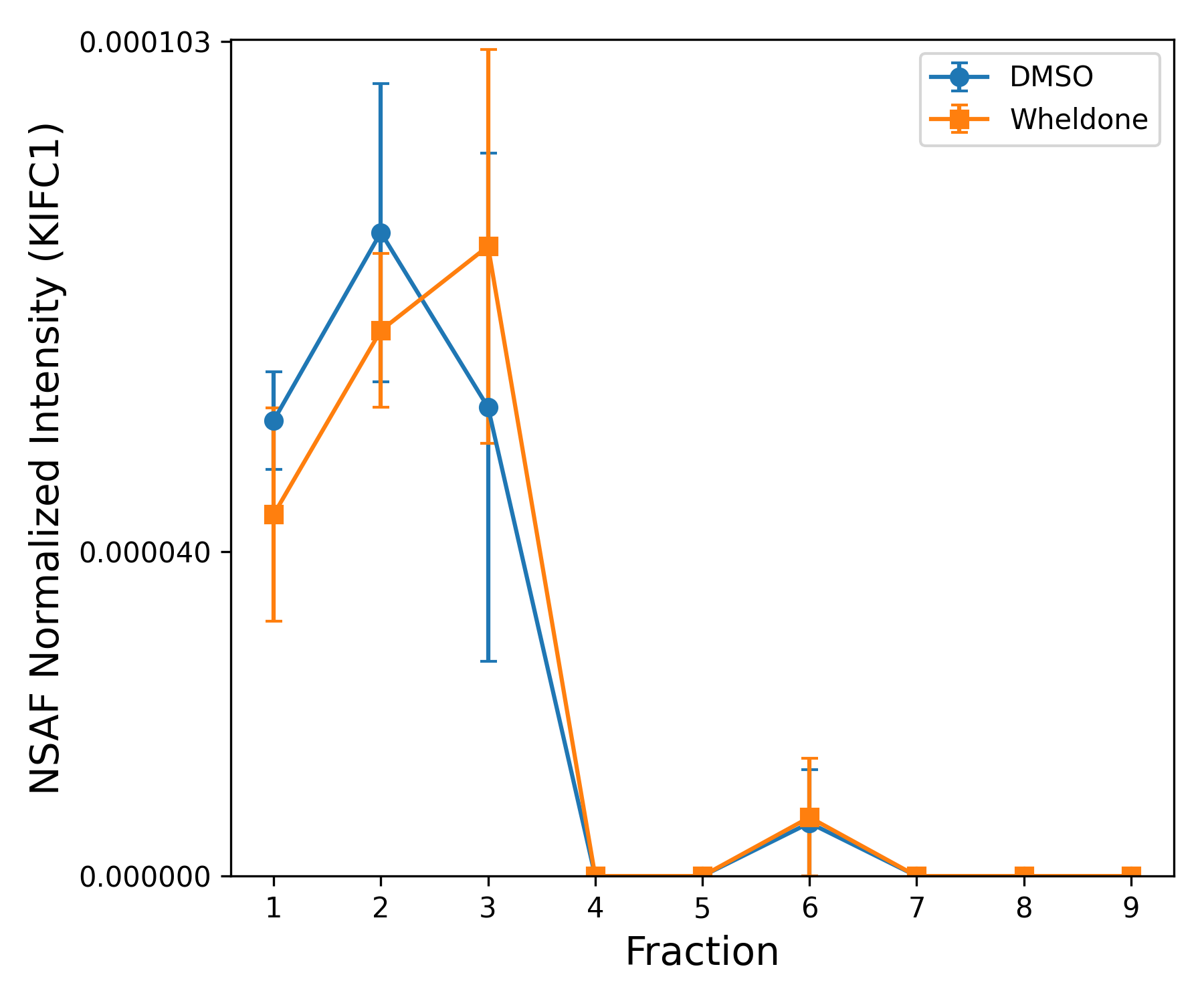

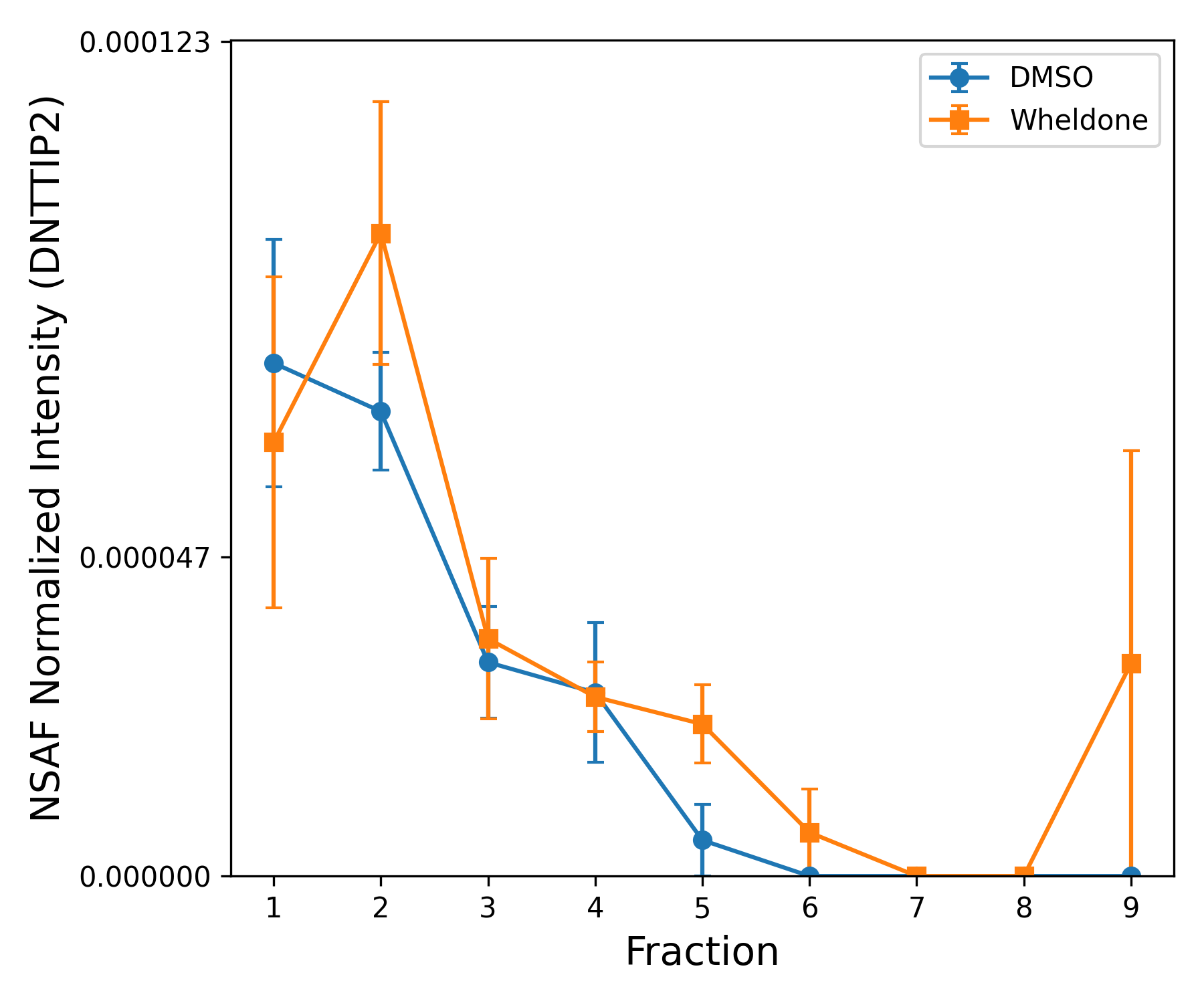

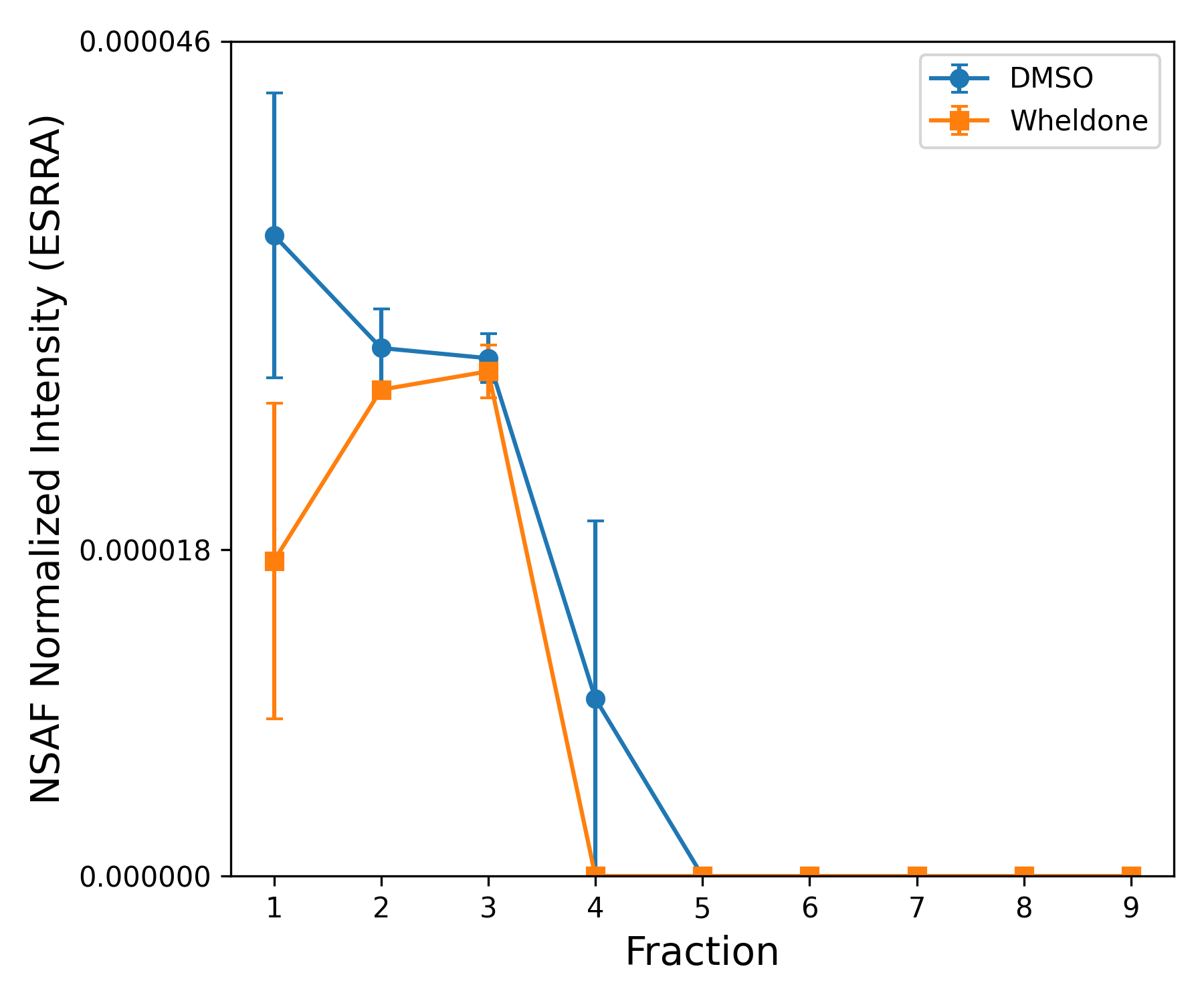

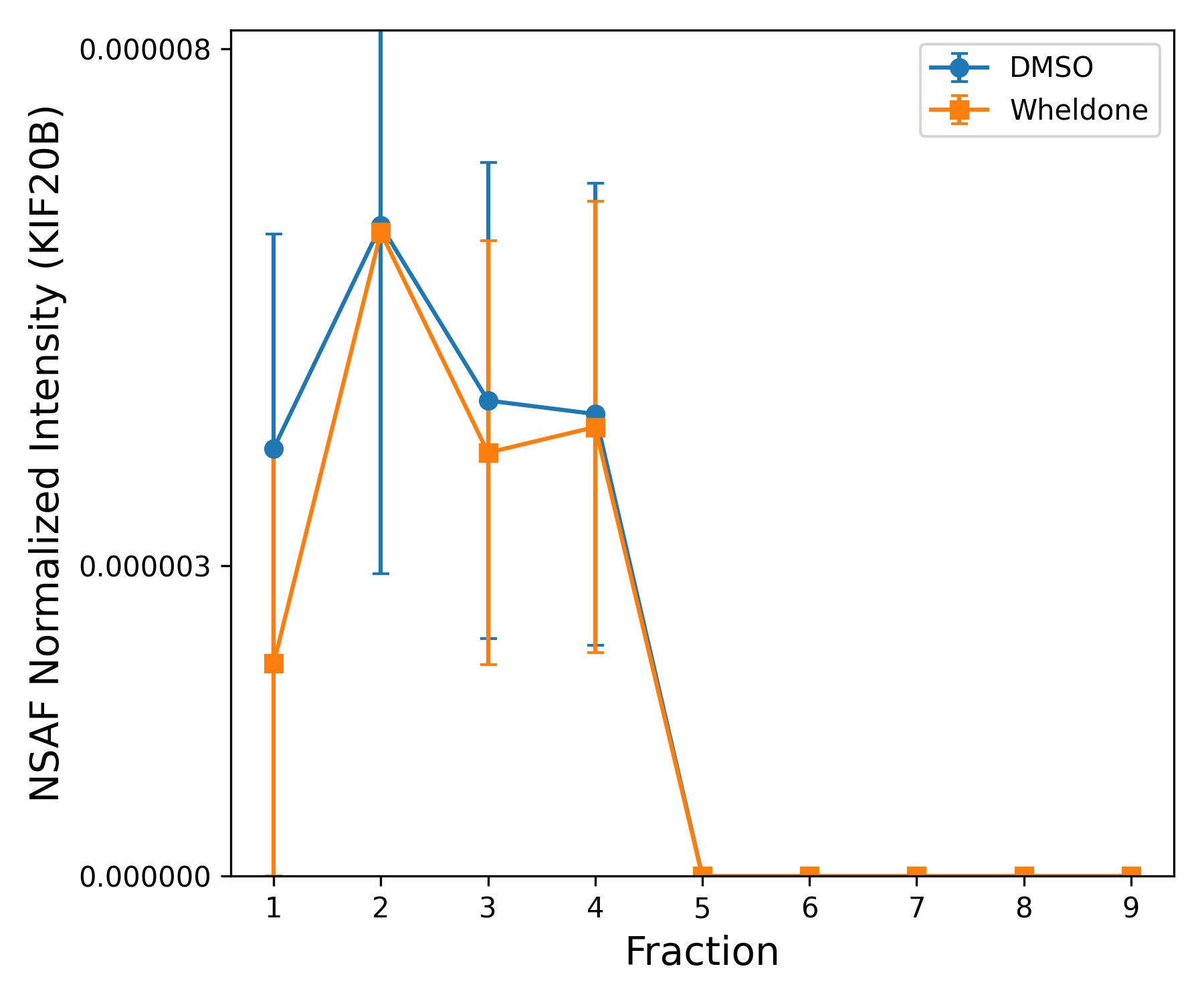

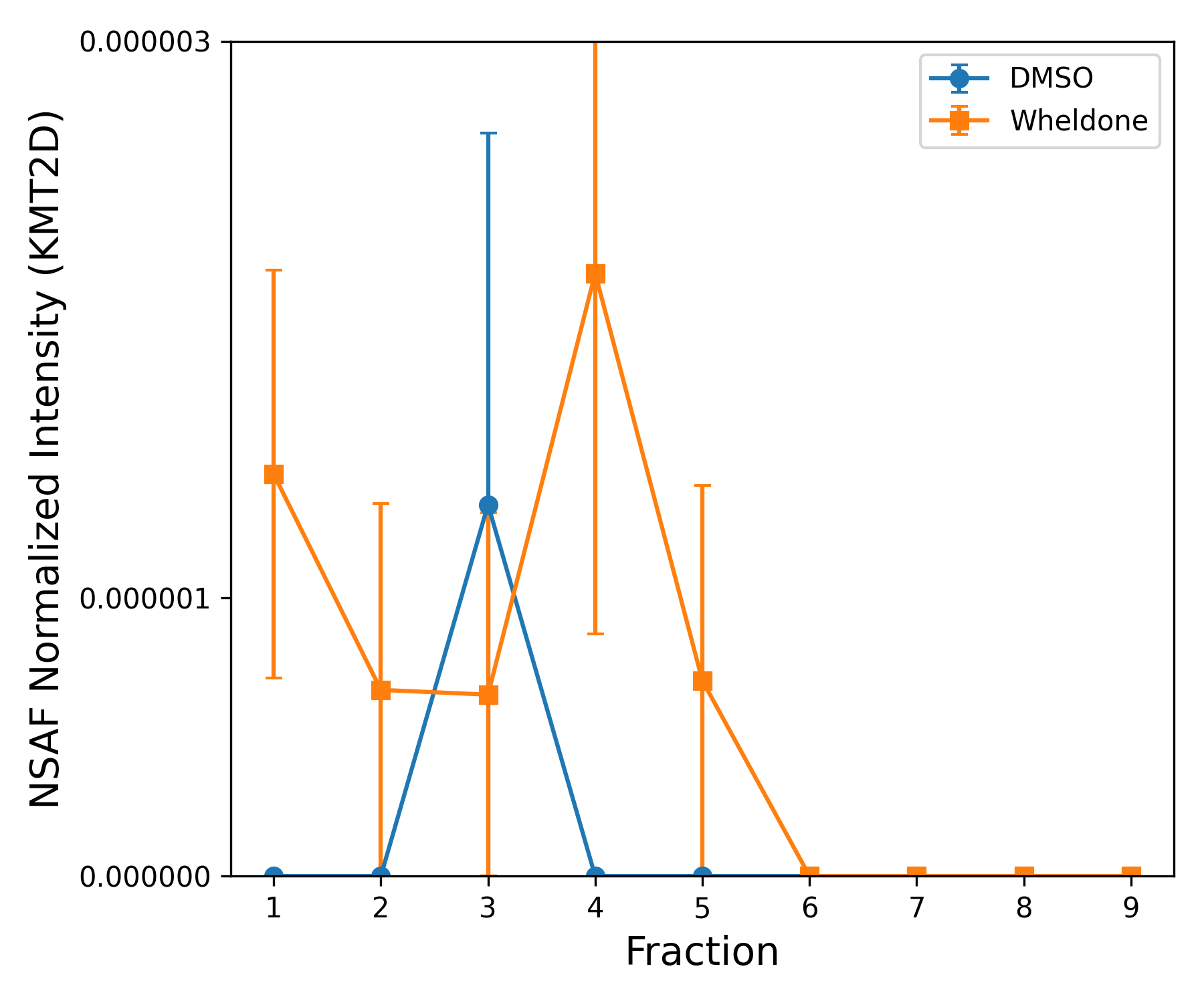

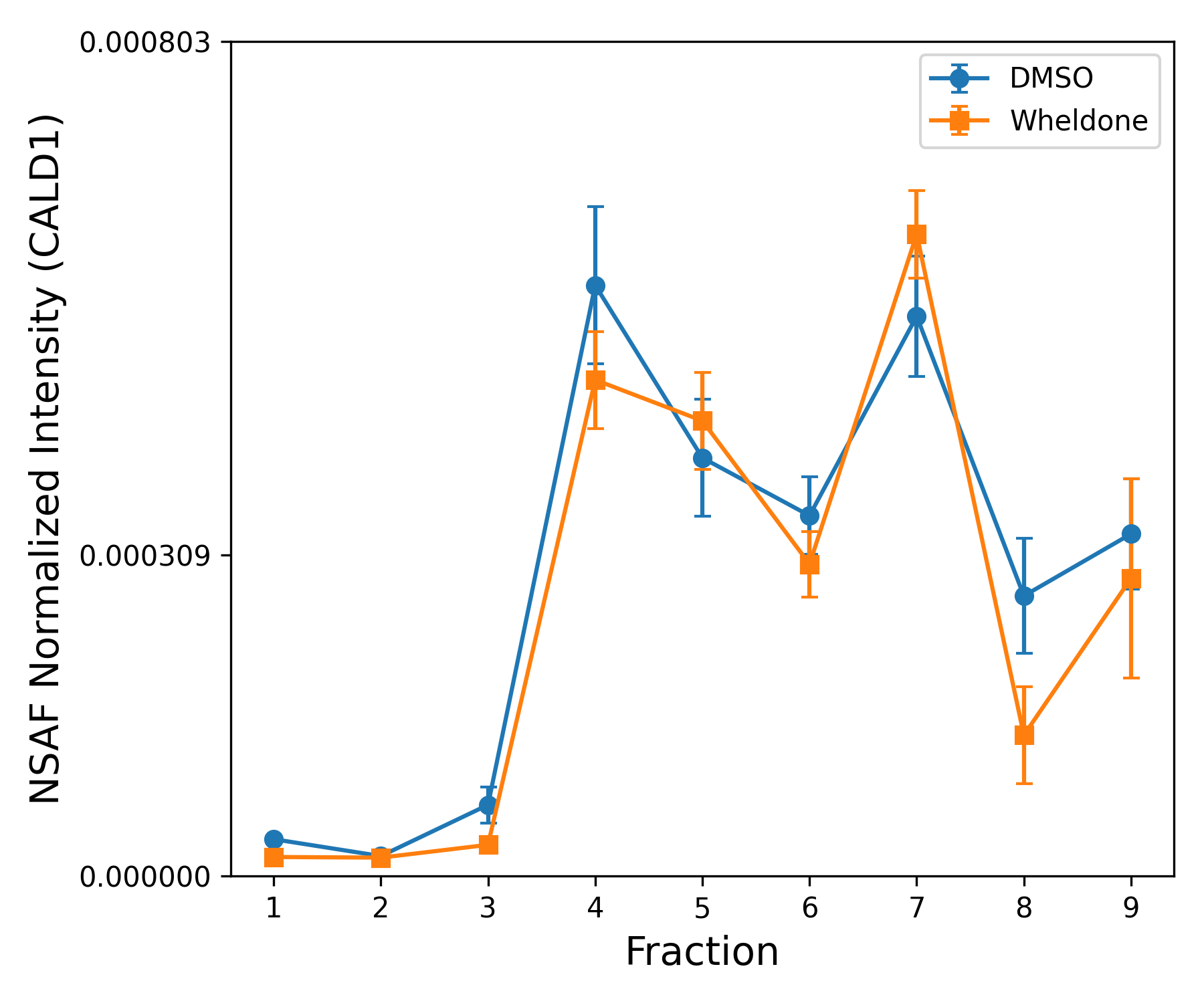

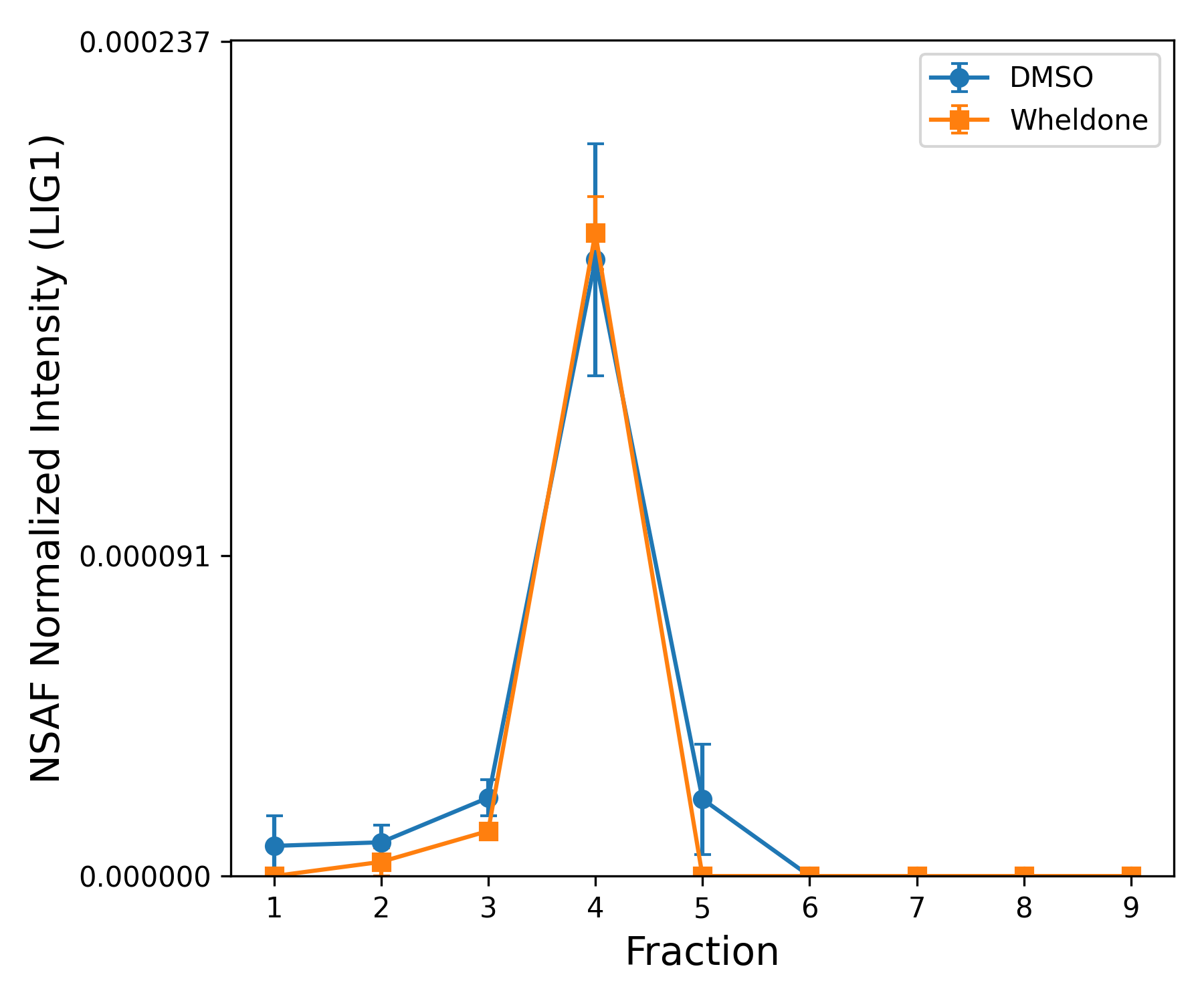

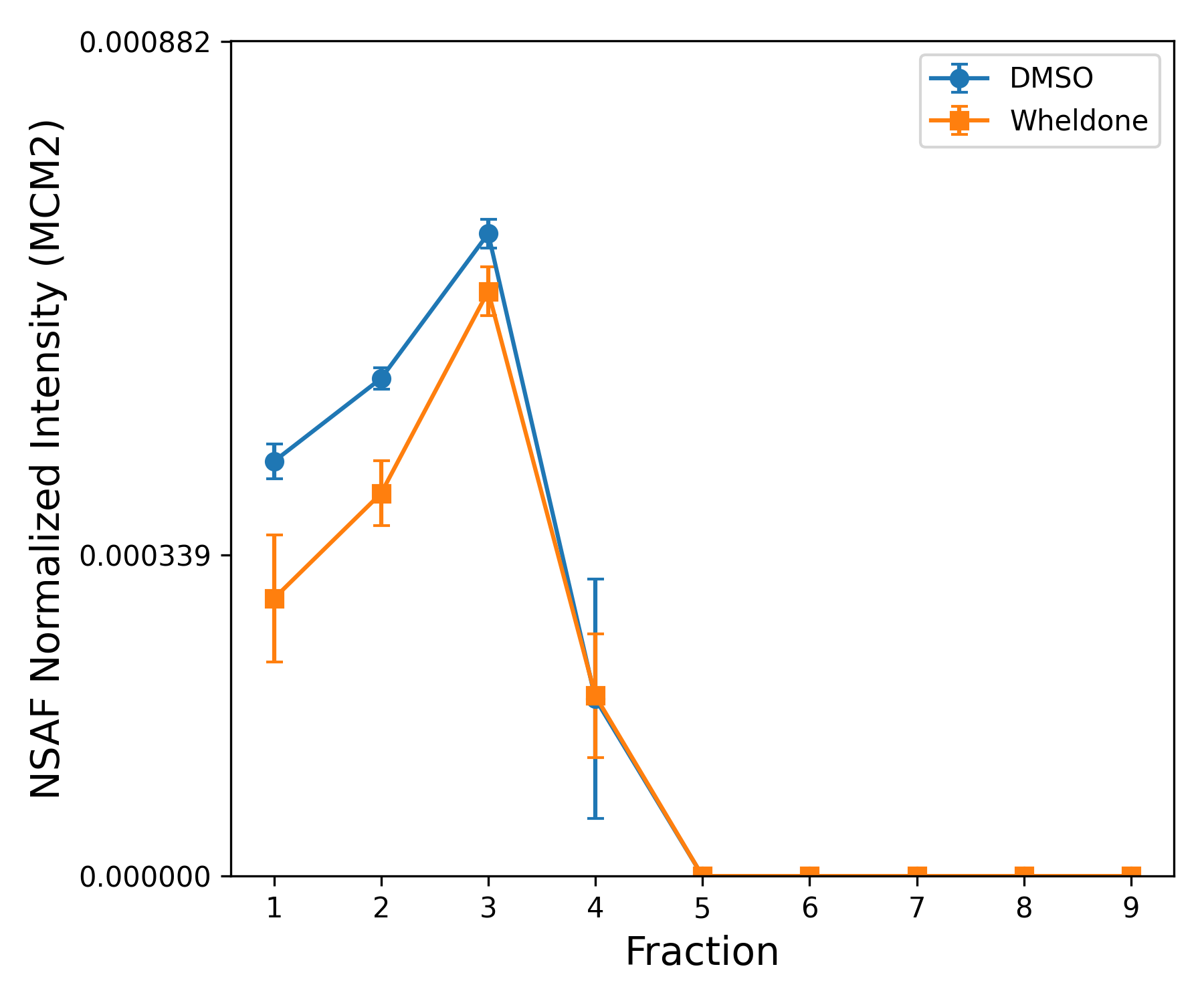

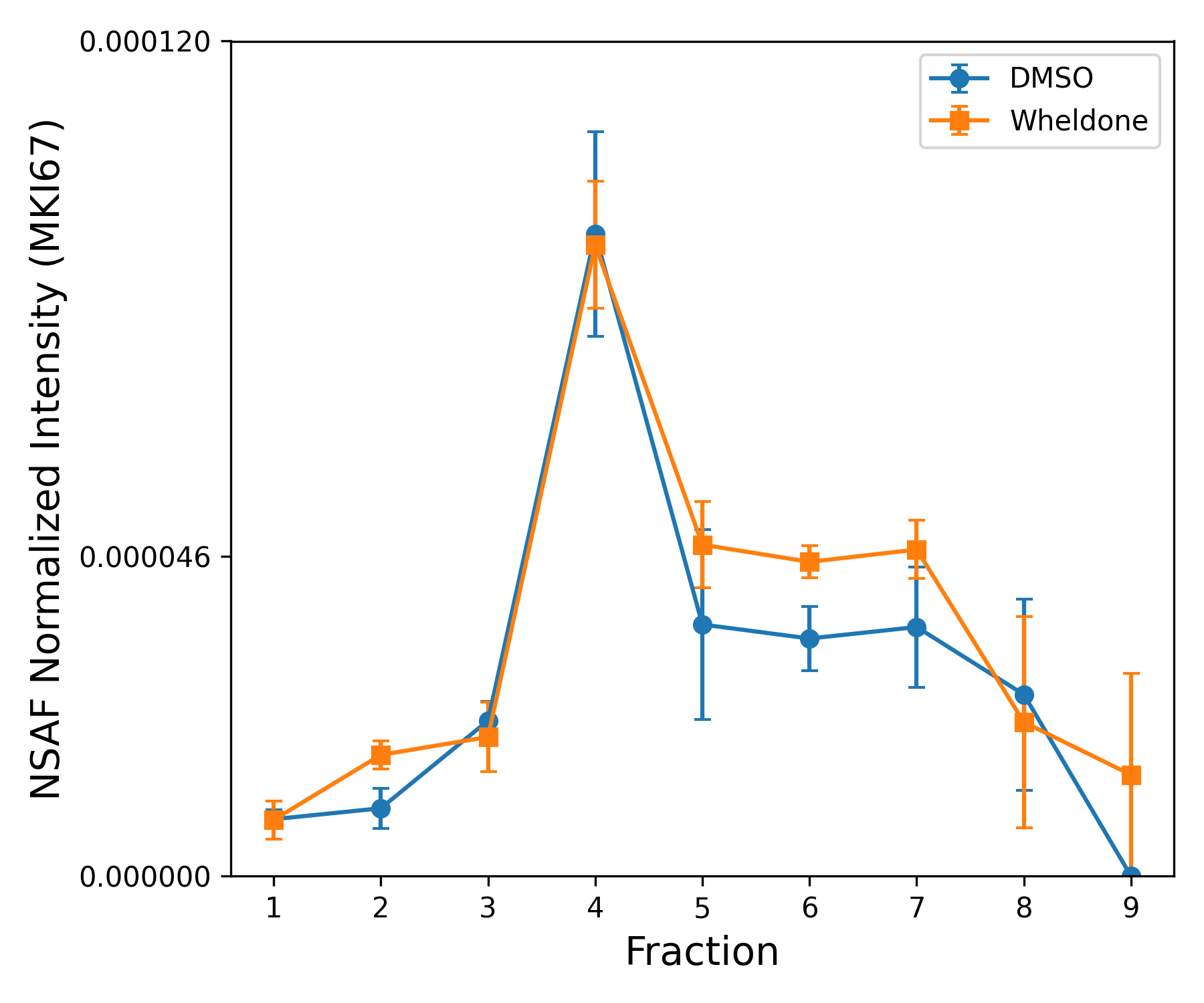

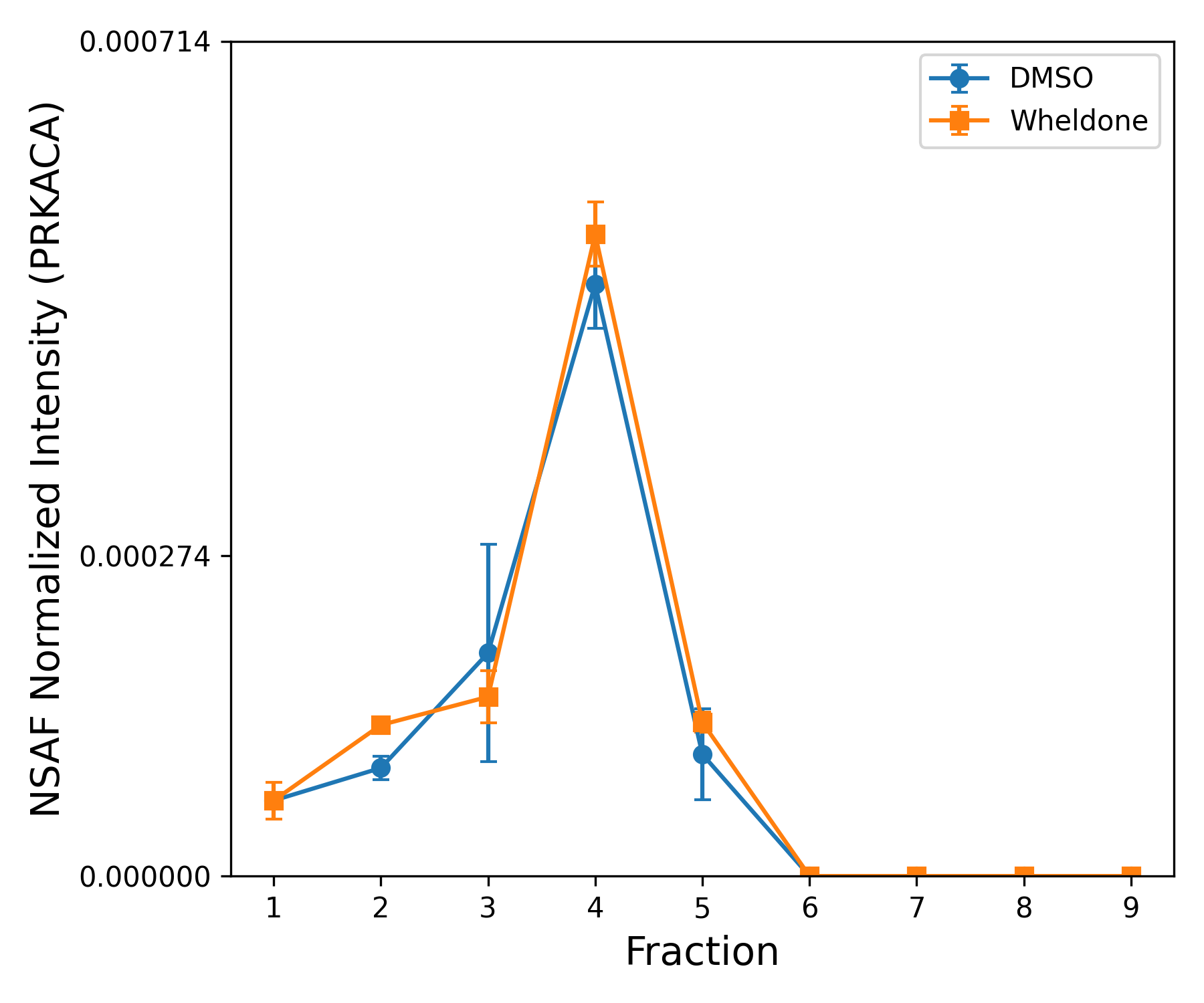

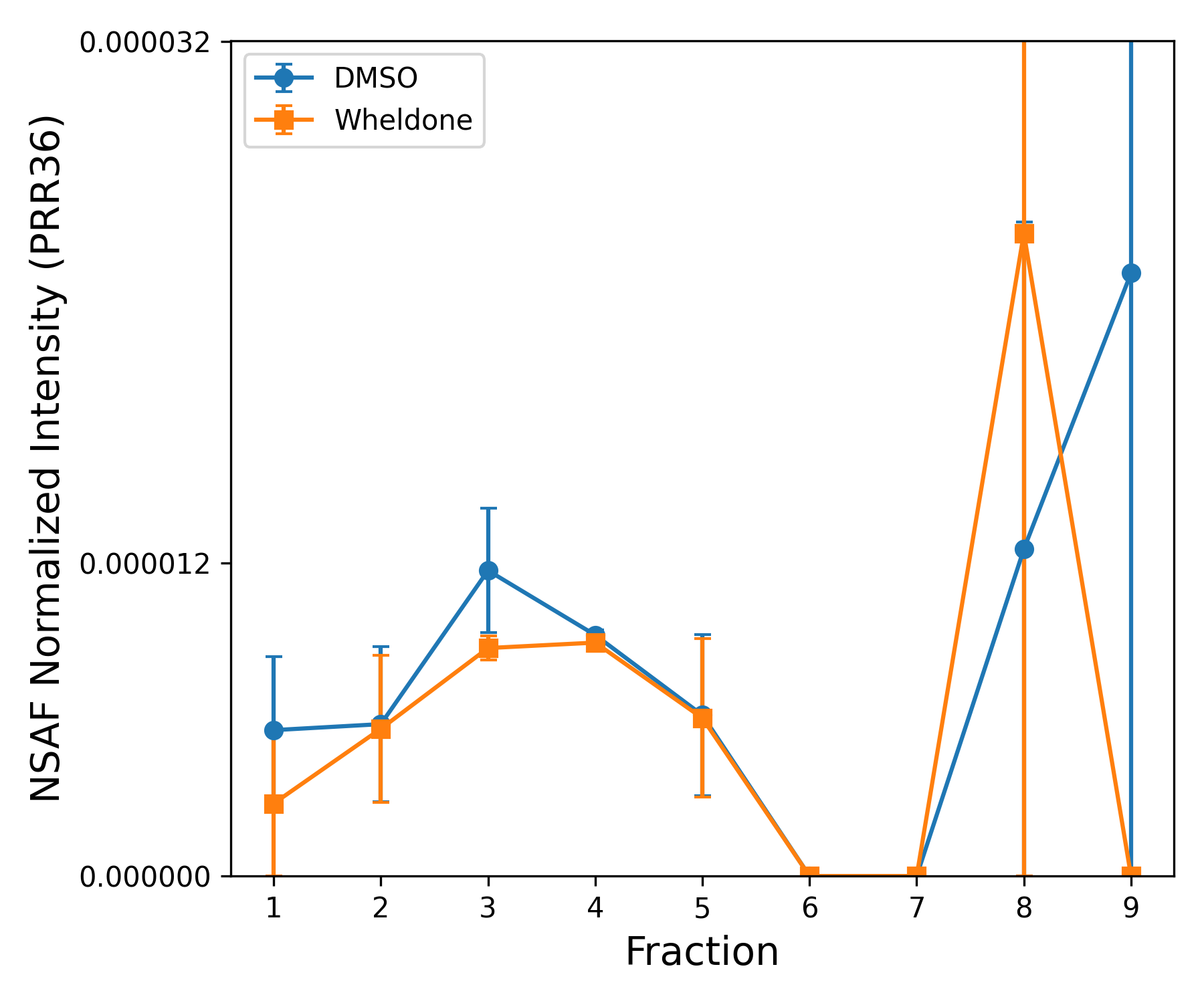

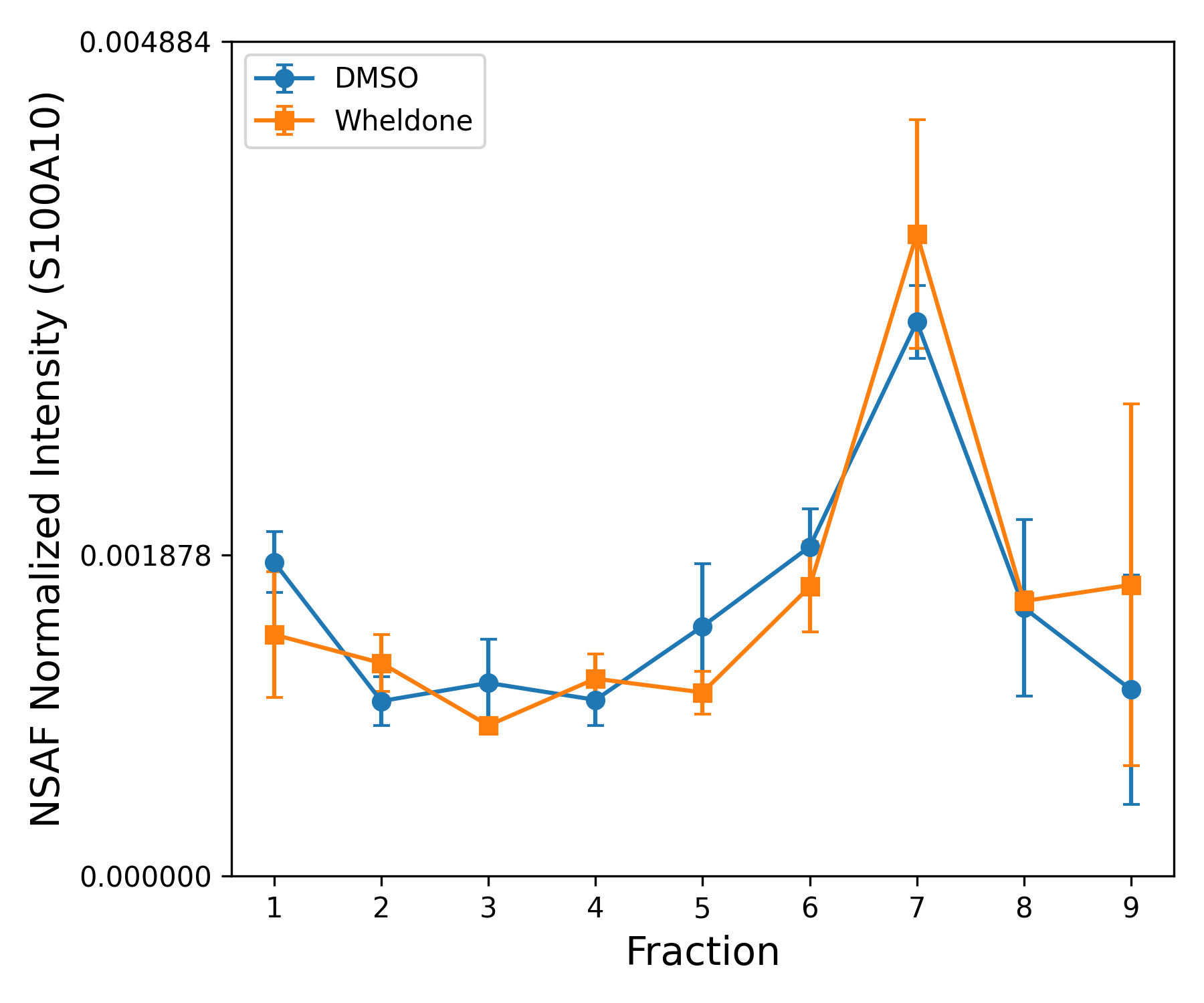

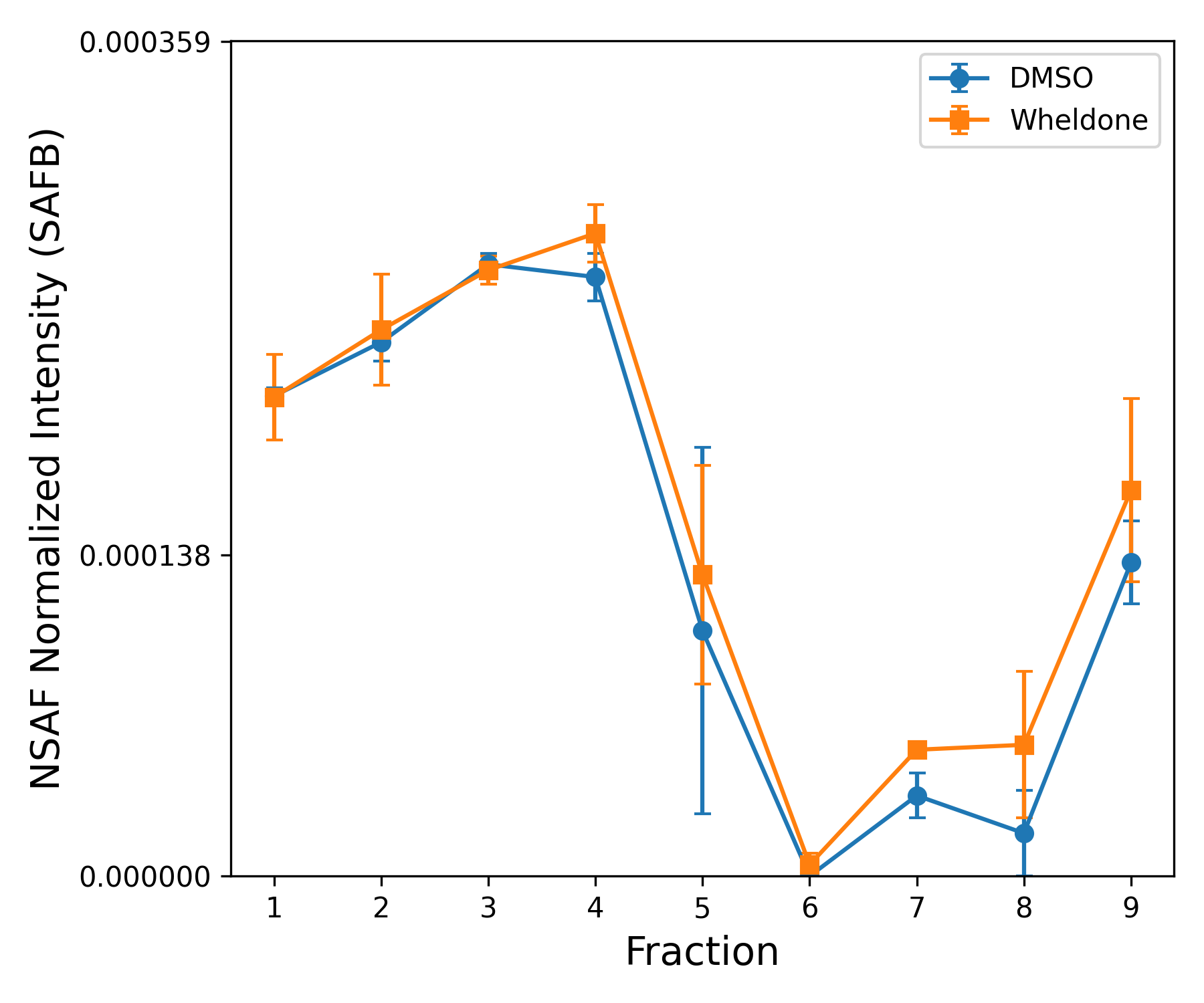

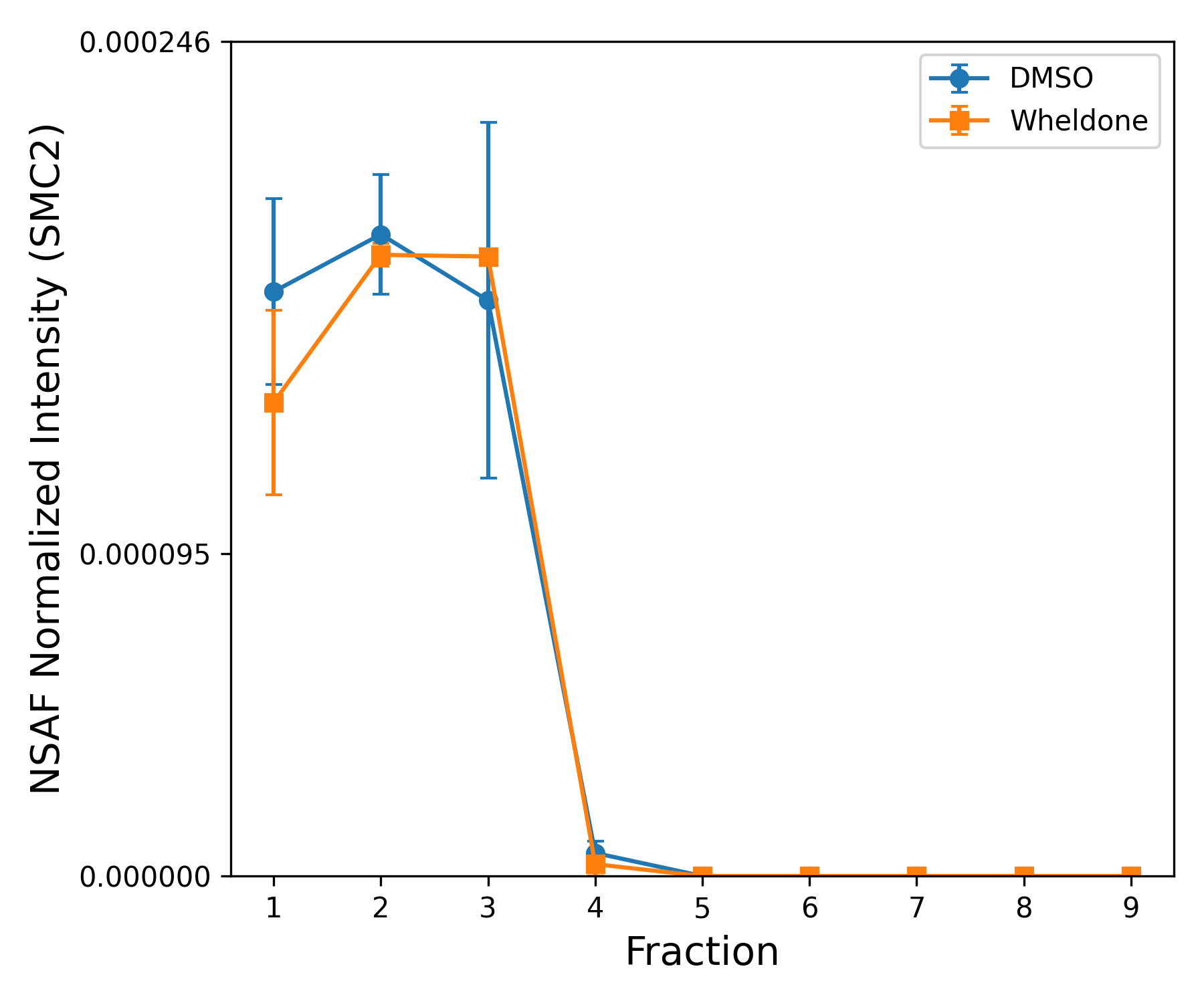

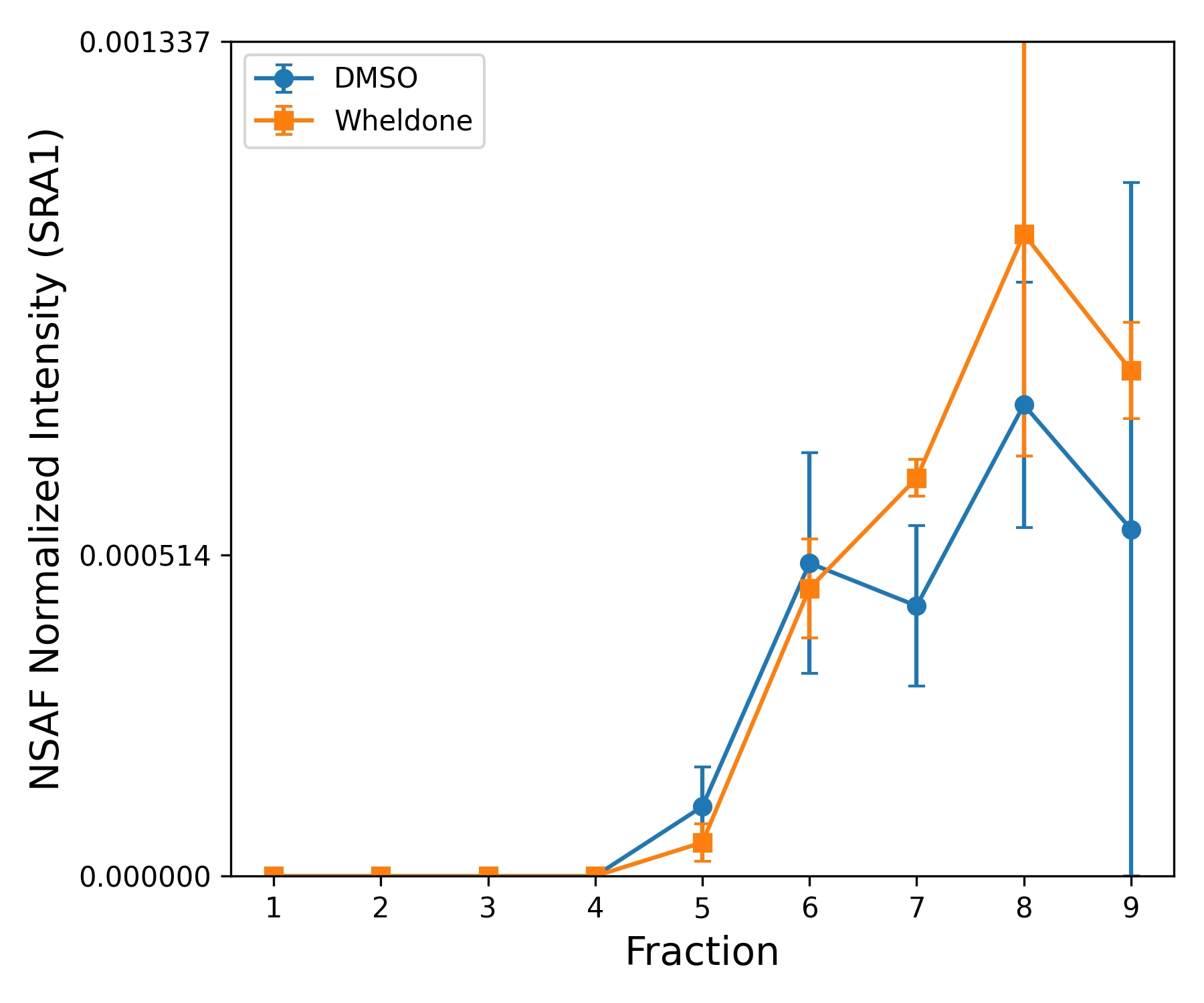

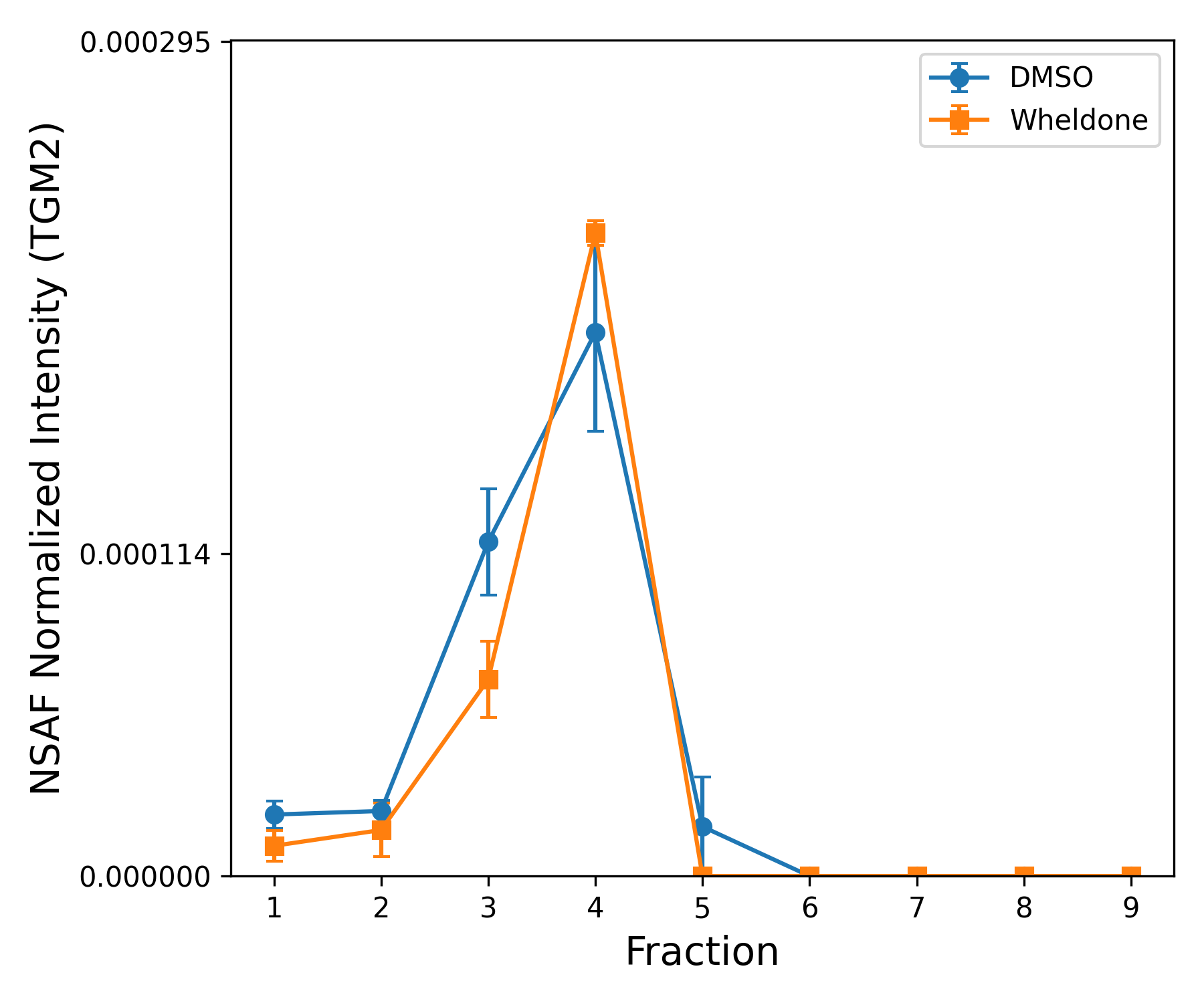


**Supplemental Figure 1. Differential precipitation of proteins analysis D in HGSOC cell lines.** Differential precipitation of proteins was performed in OVCAR3 cells. NSAF-normalized intensity of KIF11 across fractions 1-10 was compared in vehicle (DMSO)-treated and wheldone-treated cells, using GAPDH as the loading control. KIF11 levels for wheldone-treated cells peak at fraction 3, while KIF11 levels for DMSO-treated cells peak at fraction 2, demonstrating the shift in protein precipitation.


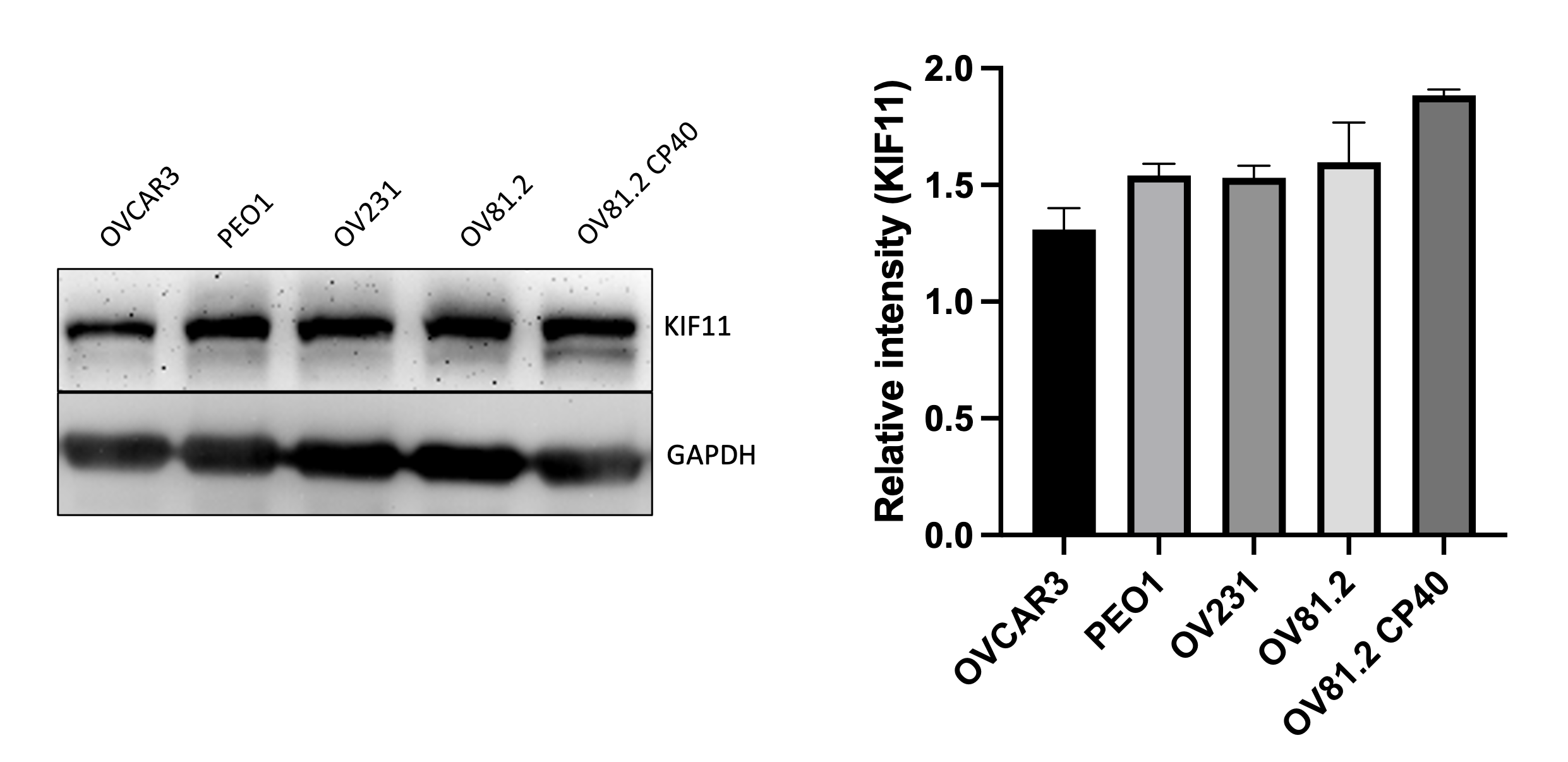


**Supplemental Figure 2. Protein expression of KIF11 in HGSOC cell lines.** Whole cell lysates from the HGSOC cell lines were collected and analyzed via western blotting for KIF11. GAPDH was used as the loading control.


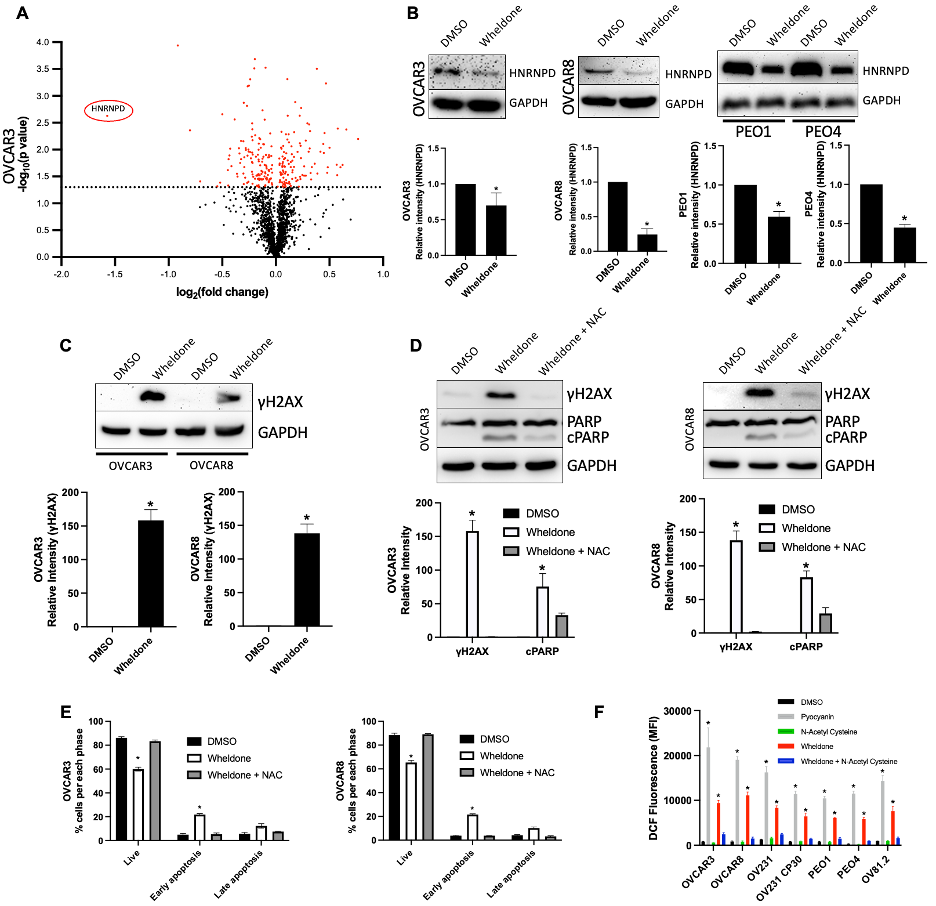


**Supplemental Figure 3. Protein expression of HNRNPD in HGSOC cell lines.** **A.** Proteomics from vehicle (DMSO)-treated or wheldone (1 µM)-treated OVCAR3 cells. Red dots represent the most deregulated proteins by wheldone treatment. **B.** OVCAR3, OVCAR8, PEO1, and PEO4 cells, treated with vehicle (DMSO), or wheldone (1 µM) for 24 hours. Representative western blots show a decrease in HNRNPD after the wheldone treatment and GAPDH serves as a loading control. **C, D.** OVCAR3 and OVCAR8 cells, treated with vehicle (DMSO), wheldone (1 µM), or combination of N-acetyl-1-cysteine (NAC) (1 mM) with wheldone (1 µM) for 24 hours. Representative western blots show an increase in γH2AX and cPARP after the wheldone treatment and GAPDH serves as a loading control. **E.** OVCAR3 and OVCAR8 cells were treated with vehicle (DMSO), wheldone (1 µM), or combination of N-acetyl-1-cysteine (NAC) (1 mM) with wheldone (1 µM) for 24 hours and stained with annexin V-FITC (AV) and propidium iodide (PI). Percentages of non-apoptotic cells, early and late apoptotic cells were quantified. **F.** OVCAR3, OVCAR8, OV231, OV231 CP30, PEO1, PEO4 and OV81.2 cells were treated with vehicle (DMSO), pyocyanin (1 mM), N-acetyl-l-cysteine (1 mM), wheldone (1 µM), or combination of N-acetyl-1-cysteine (NAC) (1 mM) with wheldone (1 µM) for 24 hours and then monitored for DCFDA fluorescence. Each experiment was performed in three biological replicates and data represent triplicate values ± SEM. For panels B and C, student’s t-test was used to compare to the vehicle and to generate p values (* denotes p<0.05). For panels D, E, and F, one-way ANOVA with Dunnett’s multiple comparisons was used to compare to the vehicle and to generate p values (* denotes p<0.05).

**
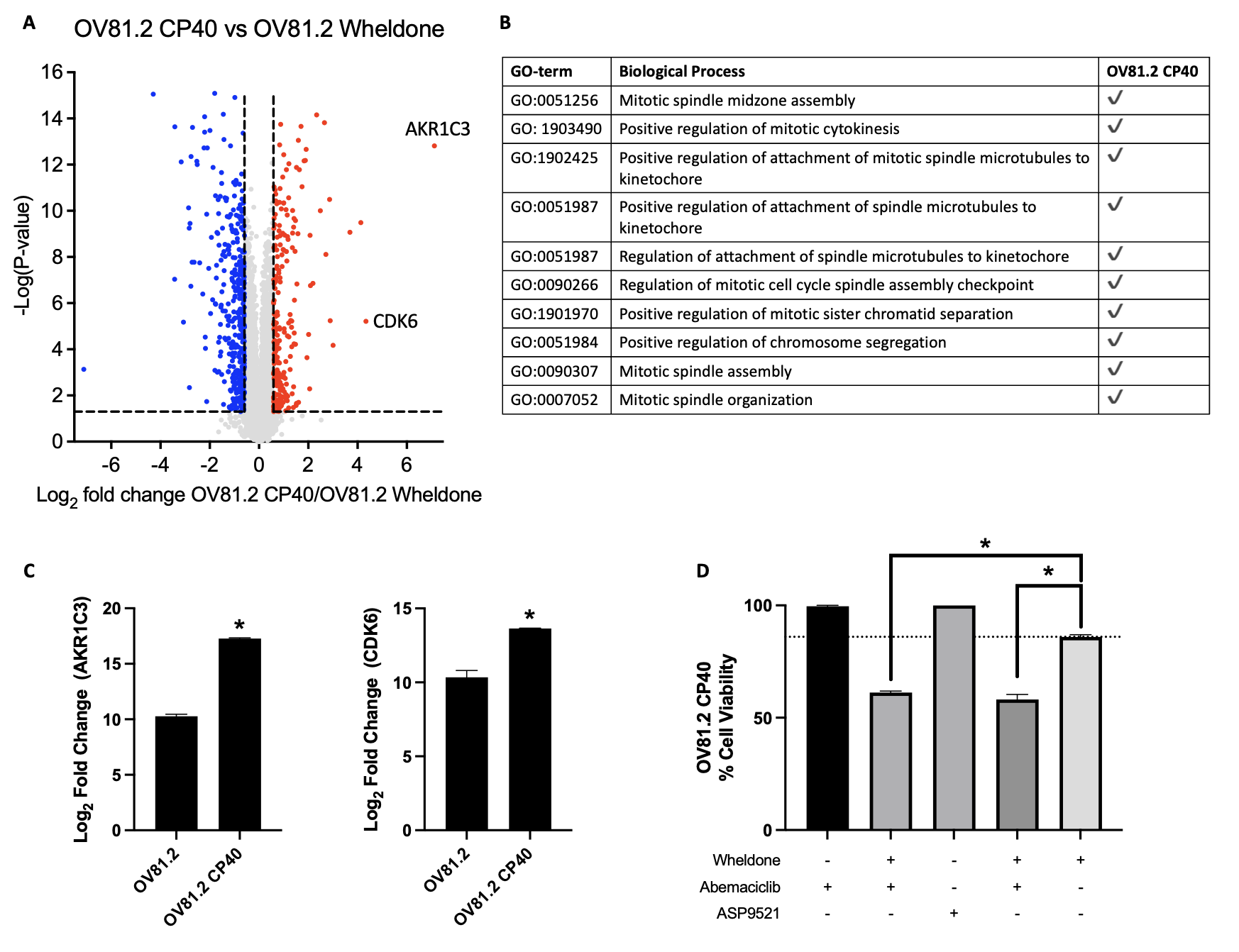
**

**Supplemental Figure 4. Wheldone, in combination with CDK4/6 inhibitor or AKR1C3 inhibitor, leads to cell death in OV81.2 CP40. A.** OV81.2 and OV81.2 CP40 cells were treated with vehicle (DMSO) or wheldone (1 µM) for 24 hours. Then, whole cell lysates were collected, global proteomics profiling was performed and compared with the vehicle. The red dots represent significantly upregulated proteins, and blue dots are significantly downregulated proteins. **B.** Gene Ontology (GO) analysis was performed on all the significantly deregulated proteins. Errors in cell cycle pathways were consistently observed in top ten deregulated pathways in both cell lines. **C.** Using the proteomics profiling results, CDK4/6 and AKR1C3 expressions were checked in both cell lines. **D.** OV81.2 CP40 cells were treated with wheldone (1 µM) or combination of wheldone (1 µM) with either abemaciclib (20 µM) or ASP9521 (30 nM) for 24 hours, using cell viability assay. Each experiment was performed in three biological replicates and data represent triplicate values ± SEM. Student’s t-test was used to generate p values (* denotes p<0.05).

| 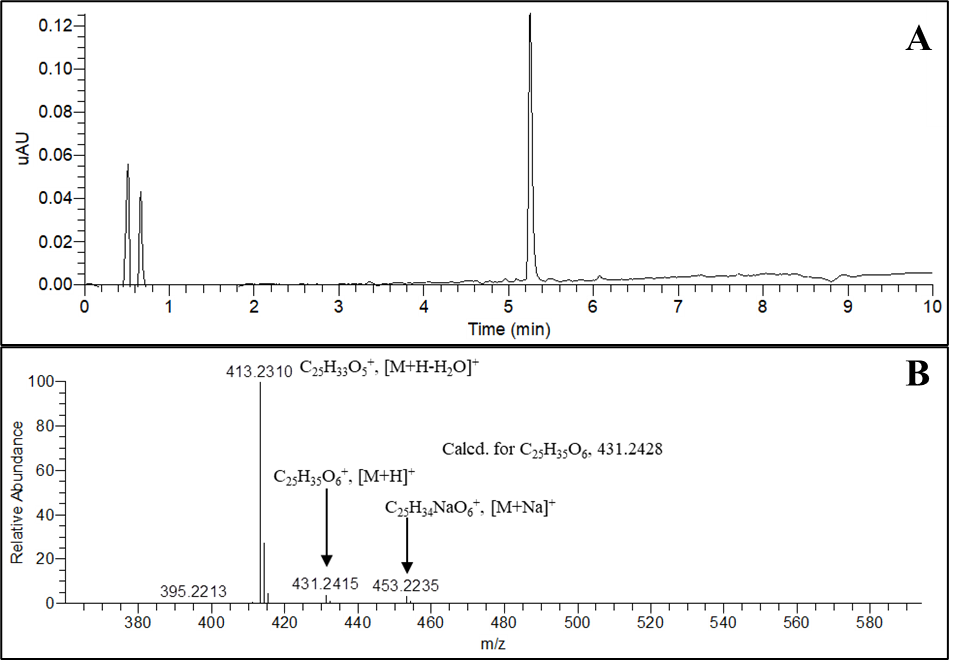 |
| --- |
| **Supplemental** **Figure 5 A**. UPLC chromatogram of wheldone, showing > 95 % purity. Data were acquired via Acquity UPLC system using an Acquity BEH Shield RP18 column (Waters, 1.7 µm; 50 × 2.1 mm) equilibrated at 40 ˚C with a flow rate set to 0.3 mL/min. The gradient system consisted of CH_3_CN/H_2_O in 0.1 % formic acid and increased linearly from 15 to 100 % CH_3_CN over 10 mins. **B.** (+)-HRESIMS spectrum of wheldone. Data were acquired using a Thermo LTQ Orbitrap XL mass spectrometer equipped with an electrospray ionization source (Thermo Fischer Scientific). The calculated value is for [M+H]^+^, indicating that the experimental data are within 5 ppm of the calculated value, per standards in the field of organic chemistry. |

| 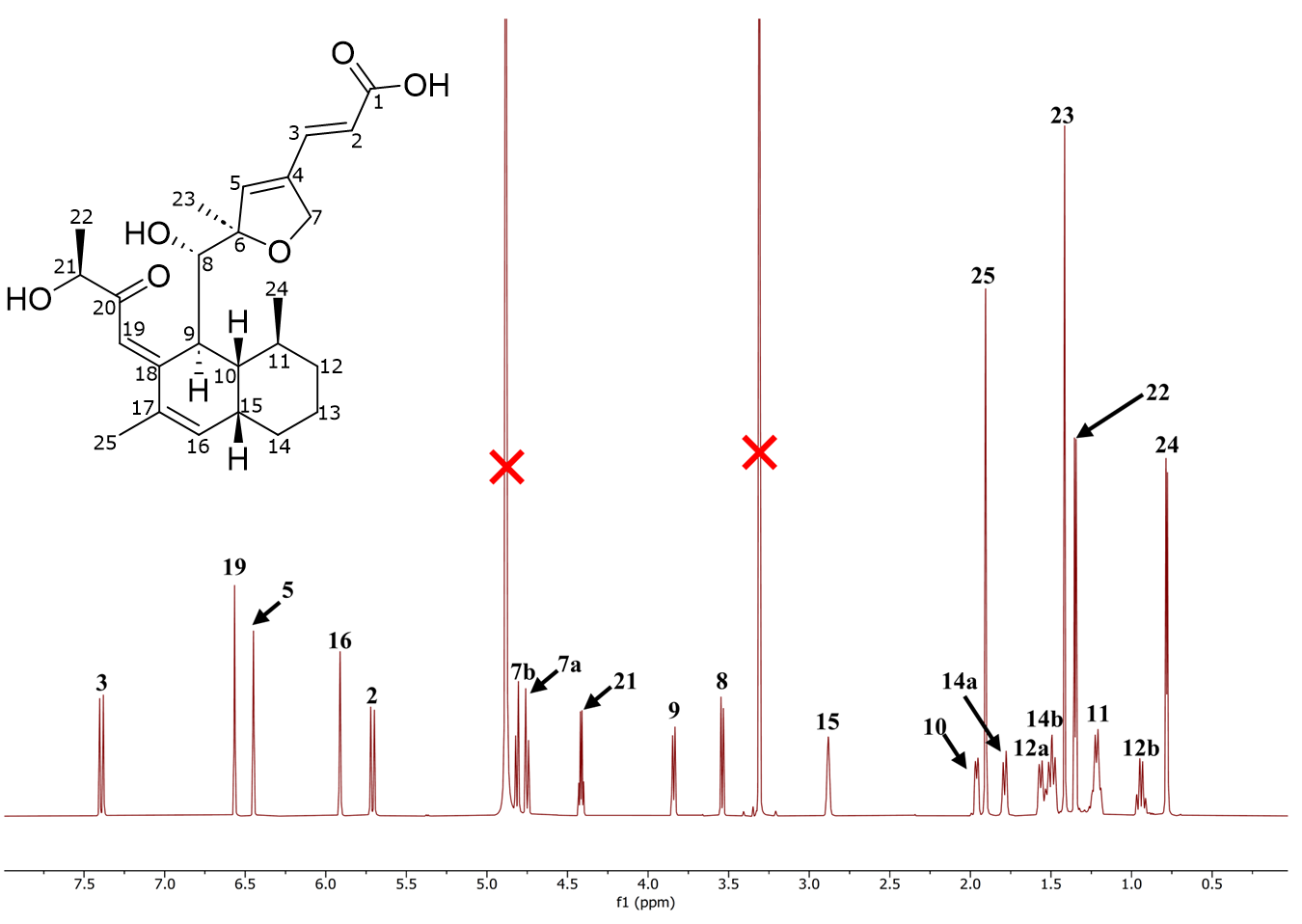 |
| --- |
| **Supplemental Figure 6** ^1^H NMR spectrum and labelled structure of wheldone in CD_3_OD [700 MHz]. |

**Table I. Primary Antibodies**

| **Antibody** | **Supplier** |
| --- | --- |
| γH2AX | Cell Signaling Technology; #2577 |
| GAPDH^a^ | Cell Signaling Technology; #2118 |
| Histone H3 | Cell Signaling Technology; #9715 |
| HNRNPD | Cell Signaling Technology; #12382 |
| KIF11 | Cell Signaling Technology; #7625 |
| PARP | Cell Signaling Technology; #9542 |
| Phosphohistone H3 | Cell Signaling Technology; #9701 |

^a^GAPDH served as a loading control.

**Table II. IC_50_ values of wheldone in HGSOC cancer cell lines.** Cells were plated and treated with wheldone, olaparib, or cisplatin for 72 hours. IC_50_ values were generated from dose-responses.

| **IC_50_ (µM)** | **PEO1** | **PEO4** | **OV81.2** | **OV81.2 CP40** | **OV231** | **OV231 CP30** |
| --- | --- | --- | --- | --- | --- | --- |
| Wheldone | 0.57 ± 0.03 | 1.37 ± 0.17 | 0.39 ± 0.01 | >40 | 0.41 ± 0.01 | 0.32 ± 0.01 |
| Olaparib | 42.40 ± 1.18 | 105.7 ± 1.5 | 24.05 ± 0.49 | 59.72 ± 2.03 | 32.16 ± 1.94 | 99.17 ± 2.13 |
| Cisplatin | 2.29 ± 0.08 | 8.78 ± 0.26 | 3.63 ± 0.10 | 8.10 ± 0.54 | 2.52 ± 0.09 | 8.85 ± 0.27 |

**Table III. IC_50_ values of abemaciclib and ASP9521 in HGSOC cancer cell lines.** OV81.2 CP40 were plated and treated with abemaciclib or ASP9521 for 72 hours. IC_50_ values were generated from dose-responses.

| **IC_50_** | **OV81.2 CP40** |
| --- | --- |
| Abemaciclib (µM) | 19.40 ± 1.03 |
| ASP9521 (nM) | 32.24 ± 7.73 |

**Table IV. Stability results of wheldone and control compound, propantheline, in human plasma**

| **Compound** | **Species** | **Concentration (µM)** | **T_1/2_ (min)** | **Remaining Percentages (%)** | | | | |
| --- | --- | --- | --- | --- | --- | --- | --- | --- |
|  |  |  |  | **0 min** | **15 min** | **30 min** | **60 min** | **120 min** |
| Propantheline | Human | 5 | 21.38 | 100.0 | 69.78 | 44.87 | 16.68 | 2.16 |
| Wheldone | Human | 5 | >511.69 | 100.0 | 105.58 | 102.79 | 97.21 | 92.45 |

Note: If the percentage of compound remaining at 120 minutes was greater than 85%, then the T_1/2_ was reported as >511.69 minutes.

**Table V. Metabolic stability of wheldone and control compound, verapamil, in pooled human and male mouse liver microsomes**

| **Compound** | **Species** | **T_1/2_ (min)** | **CL_int_ (µL/min/mg protein)** |
| --- | --- | --- | --- |
| Verapamil | Human | 7.98 | 173.68 |
|  | Mouse | 3.15 | 439.54 |
| Wheldone | Human | >255.85 | <5.42 |
|  | Mouse | >255.85 | <5.42 |

Note: For the compounds that showed an initial fast disappearance followed by a slow disappearance, only the time points that were within the initial rate were included in the calculation. If % remaining at 15 minutes was lower than 10%, then CL_int_ and t_1/2_ were reported as “>307.01” and “<4.51”, respectively. If % remaining at 60 minutes was greater than 85%, the t_1/2_ and in vitro C_Lint_ were reported as ">255.85" and "<5.42", respectively.

**Table VI. Metabolic stability of wheldone and control compound, verapamil, in pooled human and male mouse liver microsomes, with or without NADPH**

| **Compound** | **Species** | **Assay Format** | **Remaining Percentage (%)** | | | | |
| --- | --- | --- | --- | --- | --- | --- | --- |
|  |  |  | **0 min** | **5 min** | **10 min** | **20 min** | **60 min** |
| Verapamil | Human | With NADPH | 100.00 | 66.15 | 41.78 | 17.74 | 0.00 |
|  |  | Without NADPH | 100.00 | 98.32 | 102.68 | 107.38 | 99.66 |
|  | Mouse | With NADPH | 100.00 | 31.23 | 11.11 | 0.00 | 0.00 |
|  |  | Without NADPH | 100.00 | 93.36 | 104.90 | 97.20 | 103.15 |
| Wheldone | Human | With NADPH | 100.00 | 108.08 | 110.29 | 109.61 | 109.56 |
|  |  | Without NADPH | 100.00 | 93.75 | 99.22 | 95.31 | 101.56 |
|  | Mouse | With NADPH | 100.00 | 101.96 | 115.03 | 114.96 | 116.75 |
|  |  | Without NADPH | 100.00 | 100.69 | 106.90 | 104.14 | 111.03 |

**Table VII. In vivo clearance data of wheldone in female mice.** Female C57BL/6 mice (n=3/group) were given either 1 mg/kg IV or 10 mg/kg IP and plasma assessed for wheldone levels at various time points for 24 hours following dosing.

|  |  | **1 mg/kg IV** | **10 mg/kg IP** |  |  | **1 mg/kg IV** | **10 mg/kg IP** |
| --- | --- | --- | --- | --- | --- | --- | --- |
| C_0_/C_max_ | Animal 1 | 1,656 | 4,475 | CL | Animal 1 | 47.3 | NA |
| (nM) | Animal 2 | 2,007 | 3,538 | (ml/min/kg) | Animal 2 | 64.5 | NA |
|  | Animal 3 | 1,696 | 7,890 |  | Animal 3 | 38.7 | NA |
|  | **Mean** | **1,786** | **5,301** |  | **Mean** | **50** | **-** |
|  | **CV(%)** | **10.7** | **43.2** |  | **CV(%)** | **26.2** | **-** |
| T_max_ | Animal 1 | NA | 0.5 | T_1/2_ | Animal 1 | 0.68 | 1.49 |
| (h) | Animal 2 | NA | 0.25 | (h) | Animal 2 | 0.23 | 1.46 |
|  | Animal 3 | NA | 0.5 |  | Animal 3 | 1.54 | 4.30 |
|  | **Mean** | **-** | **0.4** |  | **Mean** | **0.8** | **2.4** |
|  | **CV(%)** | **-** | **34.6** |  | **CV(%)** | **81.5** | **67.5** |
| AUC_last_ | Animal 1 | 793 | 5,932 | VD_ss_ | Animal 1 | 2.3 | NA |
| (nM*h) | Animal 2 | 571 | 4,178 | (L/kg) | Animal 2 | 1.3 | NA |
|  | Animal 3 | 944 | 23,311 |  | Animal 3 | 3.8 | NA |
|  | **Mean** | **769** | **11,141** |  | **Mean** | **2.4** | **-** |
|  | **CV(%)** | **24.4** | **94.9** |  | **CV(%)** | **51.4** | **-** |
| Bioavailability |  | NA | 100% |  |  |  |  |
